# Timing Mechanotransduction: Mechanically Dynamic Biomaterials Reveal the Temporal Hierarchy of YAP/TAZ Control Nodes

**DOI:** 10.1002/advs.202515210

**Published:** 2026-03-19

**Authors:** Alessandro Gandin, Giada Vanni, Veronica Torresan, Margherita Pelosin, Rebecca Busetto, Anna Citron, Ambela Suli, Paolo Contessotto, Carlo Albanese, Francesca Zanconato, Tito Panciera, Stefano Piccolo, Giovanna Brusatin

**Affiliations:** ^1^ Department of Industrial Engineering University of Padova Padova Italy; ^2^ INSTM Padova RU, University of Padova Padova Italy; ^3^ Department of Molecular Medicine University of Padua Padova Italy; ^4^ IFOM ETS The AIRC Institute of Molecular Oncology Milan Italy

**Keywords:** cell culture, dynamic hydrogels, mechanotransduction, subnuclear adhesion, YAP/TAZ

## Abstract

Mechanotransduction is a cardinal regulator of cell behavior, yet its temporal unfolding and hierarchy remain poorly defined. Here, we develop dynamically softening polyacrylamide hydrogels that enable in situ modulation of substrate stiffness across physiological ranges while preserving integrin‐mediated adhesion. Time‐resolved analyses reveal a biphasic response to extracellular softening. YAP/TAZ are abruptly inactivated at an early stiffness threshold, coincident with rapid collapse of the subnuclear adhesion–F‐actin–LINC nucleo‐cytoskeletal continuum. At this step, peripheral focal adhesions remain unexpectedly resilient, persisting while undergoing centripetal remodeling. Disrupting SUN2 lowers the mechanosensitive threshold, whereas increased contractility raises it, still in LINC‐dependent manner. Early YAP/TAZ shutoff is accompanied by rapid microtubule reorganization away from a centrosomal aster, and by AMOT accumulation. Changes in nuclear flattening, cell rounding, and peripheral adhesion collapse emerge later at lower stiffness thresholds. Mechanotransduction is directionally asymmetric when cells are challenged in situ: YAP/TAZ switch off abruptly at a defined softness threshold, whereas reactivation is efficiently achieved only by cyclic (not static) strain, consistent with ratchet‐like temporal integration. Together, these findings establish a spatiotemporal framework for dynamic mechanotransduction and prioritize the nodes that operate on physiologically relevant timescales, providing timing‐based constraints to distinguish initiating events from downstream adaptations.

## Graphical abstract

This work develops dynamically softening polyacrylamide hydrogels for time‐resolved imaging during continuous mechanical transitions. The study revealed that mechanotransduction is biphasic; YAP/TAZ inactivation is driven by early loss of the nucleocytoskeletal continuum connecting subnuclear adhesions, F‐actin, and the nuclear envelope, coupled with microtubule reorganization and early AMOT accumulation. Changes in cell/nuclear shape and peripheral adhesions occur only as later response. Created with Biorender.com.

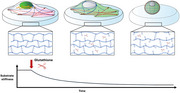

## 1 Introduction

Mechanotransduction – broadly defined as the process by which cells convert mechanical cues from their environment into biochemical signals – is a fundamental driver of cell behavior, influencing everything from migration and differentiation to tissue homeostasis and disease progression [1, 2]. In recent years, interest in mechanosignaling has expanded dramatically, alongside a growing appreciation that mechanical inputs are pervasive in vivo [1, 3]. Mechanically responsive elements are potentially numerous, including multiple adhesion subtypes, organelles and cytoskeletal networks [4, 5, 6]. This explosion of activity has also brought to the forefront a practical challenge: as the list of potentially mechanoregulated molecules and events continues to grow, it becomes increasingly difficult to distinguish primary mechanosensory steps that are immediately and directly transduced to affect cell fate, from downstream, reinforcing, or parallel responses. In this vein, a persistent “black box” remains regarding the timing, hierarchy, and thresholds with which mechanical information is first sensed and thus the means by which these are propagated in the cell. Resolving ‘where forces act, when they act, and through which structural hierarchy they are routed’ becomes essential to identify which pathways are positioned to drive nuclear transcriptional responses.

Only by following the natural unfolding of a mechanical transition in situ, and ideally live in adherent cells, can one identify which adhesion population or structural axis responds first, and which changes follow as consequences once the cell has already begun to remodel. Clearly, perturbation experiments remain essential to establish functional requirements, yet they can themselves reshuffle dynamics and obscure the native order of events; in other words, without a temporal reference frame, phenotypes produced by perturbations may conflate direct effects with indirect compensations or long‐term adaptations of cytoskeletal structures and organelles. Thus, time‐resolved analyses provide the necessary framework in which targeted perturbations can be interpreted, allowing meaningful assignment of hierarchy among distinct mechanosignaling pathways.

Addressing this timing gap in mechanotransduction has been so far challenging for at least two main reasons. First, defining the start and end of a given mechanical stimulus is rarely straightforward. In typical mechanobiology setups, cells are passaged, trypsinized, placed in suspension, and then plated on substrates of different stiffnesses or confined adhesive areas [6, 7]. These settings have so far limited the ability to follow how cells adapt to dynamic mechanical transitions. This experimental logic also contrasts sharply with in vivo conditions, where cells remain continuously attached to the extracellular matrix or basement membrane and experience gradual or sustained changes in force, due to tissue strain, damage, ECM remodeling, cell death, proliferation, and myriads other processes [1]. For example, in situ changes in extracellular mechanics are known to drive cellular reprogramming in the context of development, regeneration, and cancer [2, 8, 9, 10, 11, 12], while cells remain adherent to their substrate.

Second, resolving timing is complicated by the choice of mechanotransduction readout. Many studies rely on phenotypic outcomes such as growth or differentiation, which are slow processes occurring over hours to days, and integrating multiple signaling pathways [13, 14]. Inevitably, long‐term responses are intrinsically confounded by feedback and adaptation, including the fact that cells actively remodel ECM composition and mechanics (inside‐out signaling) [5], thereby continuously changing the very mechanical input being interrogated. These features obscure the immediate events that initiate mechanosignaling and make it difficult to pinpoint when, and through which structural routes, the genome first receives the mechanical message.

Together, the above challenges underscore the need for analytical tools that recapitulate physiologically relevant mechanical transitions under persistent adhesion and provide fast, direct readouts of mechanical signaling compatible with minute‐scale resolution. With this background in mind, we aimed to overcome existing limitations by developing new biomaterials that allow to visualize dynamic mechanosensing in living cells. Here, we used nuclear vs. cytoplasmic localization of the universal mechanotransducers YAP/TAZ transcriptional regulators as beacon to visualize effective transmission of mechanical information down to gene‐expression changes [1]. Our findings revealed that mechanosignaling occurs through at least two distinct steps, a first one closely associated to nuclear vs. cytoplasmic localization of the YAP/TAZ transducers, and a later step, primarily concerned with the restructuring of the cellular adhesive architecture and nuclear shape.

## 2 Results

### 2.1 Synthesis of Adhesive Hydrogels Amenable to In Situ Softening

**FIGURE 1:**
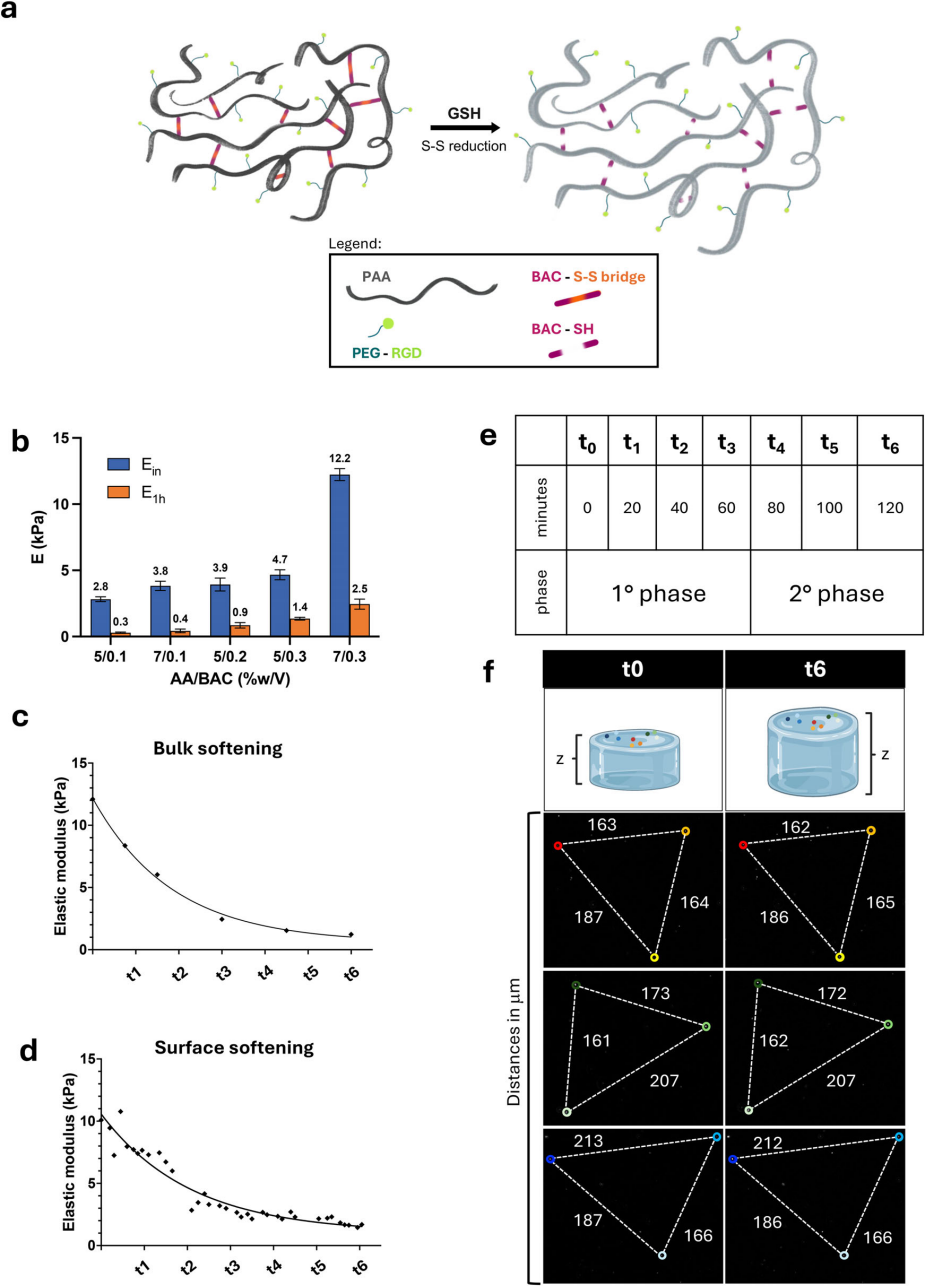
(a) Scheme of the DPAA gel and of its softening by degradation of S–S bridges with the GSH addition. For the synthesis, glass coverslips were functionalized with methacrylate moieties to covalently bind hydrogels on the surface. Acrylamide (AA), bisacryloyl‐cystamine (BAC) and a PEG monomer conjugated with a RGD containing peptide are copolymerized with a radical reaction. Different ratio between AA and BAC are used to achieve tunable initial stiffness in the range of about 3–12 kPa (Table S2). All chemical compounds used are reported in Table S1. (b) Initial and final stiffnesses of gels with different formulations degraded for 1 h with 1 mm of GSH (see details in Table S2). (c) Bulk stiffness measured at different time points during softening with 1 mm GSH of a gel with initial stiffness of about 12 kPa (see details in Table S3). (d) Surface stiffness measured with AFM at different time points during softening of a gel with initial stiffness of about 12 kPa. (e) Time window – timepoints correlation. Biphasic behavior shows a first phase corresponding to softening between t_0_ and t_3_ and a second phase extending between t_4_ and t_6_. (f) Lateral swelling behavior investigated by confocal microscopy on gels embedding fluorescent particles (diameter 0.5 µm). Lateral swelling is measured monitoring the variation of their distance during 2 h softening (1 mm GSH). Gels have a diameter of 15 mm and a thickness of less than 1 mm. Covalent anchoring to the glass substrate restrict lateral expansion, allowing swelling only along the *z*‐axis. Gels drawing is a schematic representation and is not to scale.

To investigate the timing of cellular mechanotransduction from the extracellular matrix (ECM) to the nucleus, we developed a cell‐compatible hydrogel system that permits rapid, in situ modulation of substrate stiffness. This design allows cells to remain continuously attached to a substrate that progressively softens. For hydrogel composition, we selected polyacrylamide (PAA) substrates functionalized with RGD adhesive peptides. PAA's chemical tunability, well‐established fabrication protocols, and widespread popularity for mechanobiology studies, make it an ideal choice for precise control over mechanical properties [7, 15], while the presence of RGD ensures reliable, integrin‐mediated cell adhesion. To impart dynamic control over stiffness, we envisioned an innovative procedure: during gel synthesis, we incorporated disulfide (S–S) crosslinkers (Figure 1a and Table S1) which can be cleaved to thiols (SH) through reduction. For this reduction step, we opted to use glutathione (GSH), a naturally occurring molecule that is maintained at high millimolar concentrations (up to 10 mm) inside cells to prevent oxidative damage, yet exists at negligible levels extracellularly [16]. Because GSH is cell‐impermeable, adding even a low‐concentration spike of it to the culture medium provides a highly effective “switch” to start softening the disulfide‐crosslinked PAA – hereafter referred to as dynamic PAA (DPAA) hydrogels.

DPAA was synthesized by radical copolymerization of various formulations of acrylamide (AA) and bisacryloyl‐cystamine (BAC) in water (Figure 1b and Table S2). For RGD functionalization, we first conjugated a maleimide‐PEG‐acrylate monomer with a short RGD peptide, creating an acrylate‐modified adhesive peptide (AC‐PEG‐RGD). Then, AA and BAC were co‐polymerized with AC‐PEG‐RGD, each at fixed concentration of 3 mm for all gels (see details in Tables S2). The introduction of a PEG chain was to overcome a known limitation of PAA hydrogels functionalized with adhesive peptides, that is the presence of PAA “brushes” on the gel surface, which inhibit the availability of adhesive sequence to cellular integrin receptors [17].

To optimize the dynamic range of DPAA stiffness (i.e., the modulus values before and after GSH‐induced softening), we synthesized hydrogels at various initial rigidity levels by varying both monomer and crosslinker concentrations (Table S2). After 1 mm GSH was added for 1 h, we measured the modulus changes via micropipette aspiration (Table S3). Results showed that DPAA gels could be tuned to distinct starting stiffness (up to 12 kPa), which then softened by up to tenfold – for instance, dropping from 12 to 1.2 kPa or from 3 to 0.3 kPa – as such spanning the entire spectrum of physiologically relevant ECM rigidities [18] (Figure 1b and Table S2).

Next, we investigated the temporal dynamics of hydrogel softening. All DPAA gels exhibit a significant decrease in stiffness within the first hour of GSH treatment, regardless of their initial rigidity (Figure 1b). For example, a DPAA gel starting from the highest stiffness module of 12 kPa would soften down to 2.5 kPa in 60 min, and then more slowly reach 1.2 kPa during the second hour (See Figure 1c). At this point the gels reach a plateau that remains stable even upon extending the GSH exposure to 4 h (Figure S1 and Table S3). This plateau is likely attributable to PAA chain entanglements in the gel network rather than GSH depletion via oxidation, as replenishing GSH does not further reduce stiffness. We also measured mechanical changes at the surface level, as this is what cells experience. Atomic force microscopy (AFM) measurements confirmed that DPAA hydrogels undergo a comparable modulus shift and timing of softening on the adhesive surface (Figure 1d). Hydrogels are covalently anchored, at their bottom surface, to a silanized glass substrate and fabricated as thin disks (thickness <1 mm; diameter ∼15 mm). This geometry and anchoring strongly restrict lateral expansion, allowing swelling only along the z‐axis, as such limiting the possibility that reduction of hydrogel crosslinking could affect mechanosignaling indirectly, by changing the superficial density of adhesive ligands due to lateral swelling [17]. Indeed, particle tracking analyses confirmed that substrates did not exhibit substantial changes in their lateral dimensions during 2 h of softening and swell only along the *z*‐axis (Figure 1f and Figure S2).

### 2.2 Live Imaging of Adherent Cells on Softening Gels Reveals Their Biphasic Adaptation to Substrate Stiffness

Focal adhesions are the most mechanistically understood structures in mechanosignaling at the cell surface: integrin clusters that assemble into focal adhesions sense and transmit mechanical forces from the extracellular environment to the actin cytoskeleton. Actin stress fibers, together with other cytoskeletal elements including microtubules and intermediate filaments, then relay forces across the cell [5, 6]. A key outcome of this force transmission is the nuclear translocation of mechanosensitive transcription factors such as YAP/TAZ [1, 7]. Recent work, including our own, has shown that YAP/TAZ mechanoregulation is closely associated with a nucleo‐cytoskeletal continuum in which a distinct pool of ventral, nuclear‐associated actin fibers and subnuclear adhesions mechanically couple the basal adhesive interface to the nuclear envelope via the LINC complex [19, 20, 21, 22, 23]. Importantly, however, these observations were largely obtained under static conditions, and therefore could not resolve the timing, hierarchy, or directionality of events underlying the transition between mechano‐ON and mechano‐OFF states.

**FIGURE 2:**
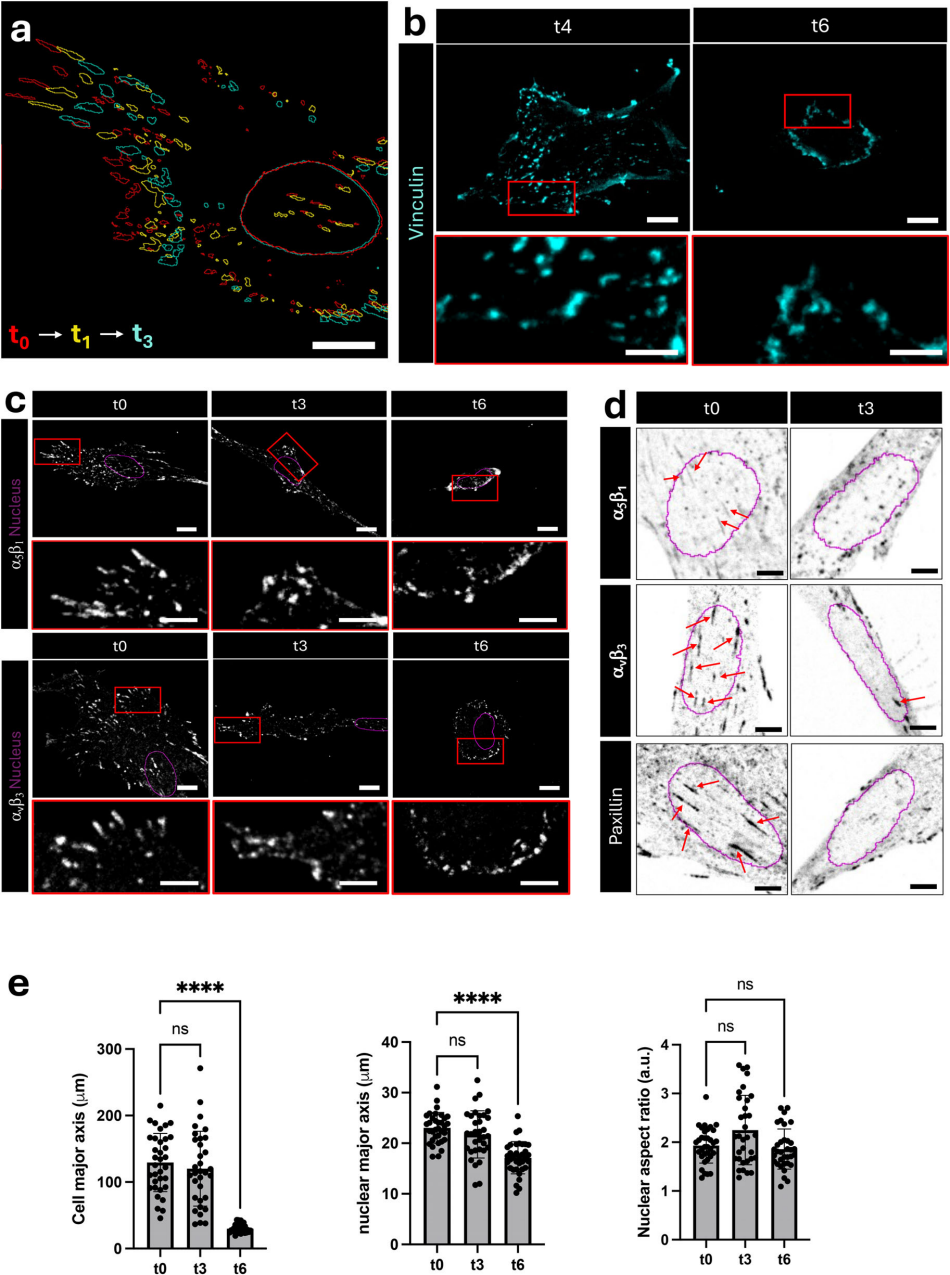
a) Still image of Movie S2 (live imaging of WI38 cells infected with Venus‐Vinculin) tracking focal adhesions during gel softening at t0, t1, t3 (in red, yellow, and cyan, respectively). Cells are seeded on gels of about 12 kPa as initial stiffness, allowed to adhere to the hydrogel surface overnight, and imaged during gel softening. Nuclear outline is obtained by Hoechst staining and tracked as focal adhesions. Scale bar 10 µm. See also Figure 2e for cell major axis quantification at the indicated timepoints of gel softening, supporting evidence from Movie S1. b) Representative images of WI38 cells seeded on 12 kPa DPAA gel and imaged during the second phase (t_4_–t_6_) of gel softening. Cells are fixed at t_4_ and t_6_ and stained for vinculin. Scale bar 10 µm for low magnification images and 5 µm for zoom‐in. c) Immunofluorescence images of α_5_β_1_ and α_v_β_3_ integrins in WI38 seeded on DPAA gels with about 12 kPa of initial stiffness and fixed at different time points during gradual softening (t_0_, t_3_, t_6_). Second and fourth rows are magnifications of selected areas (red squares) from first and third rows, respectively. Scale bars 10 and 5 µm for magnified images (*n* = 13 for t_0_, *n* = 13 for t_3_, and *n* = 10 for t_6_). d) Representative immunofluorescence images of cells on degradable hydrogel at 12 kPa (t_0_) and 2.5 kPa (t_3_) showing subnuclear cellular adhesions (identified through paxillin, α_5_β_1_ and α_v_b_3_ staining and highlighted by red arrows). Magenta circles represent the outline of the nuclear projected area. Scale bar 5 µm. e) Quantification of cell and nuclear shape of WI38 cells seeded on DPAA gels with about 12 kPa of initial stiffness at different time points during gradual softening. Cell major axis is analyzed from the fluorescently tagged phalloidin signal (*p*‐values t_0_–t_3_: 0.8345; t_0_–t_6_: <0.0001), Nuclear major axis (*p*‐values t_0_–t_3_: 0.4509; t_0_–t_6_: <0.0001) and nuclear aspect ratio (*p*‐values t_0_–t_3_: 0.0779; t_0_–t_6_: 0.8195) are analyzed from Hoechst signal. Data in (e) are reported as means and standard deviations. Statistical significance is determined with one‐way ANOVA with Welch correction. *n* = 34 for t_0_, *n* = 33 for t_3_, and *n* = 35 for t_6_. Data were tested for outliers through the ROUT method with Q = 1%.

Leveraging the dynamic mechanical properties of DPAA hydrogels, we conducted live‐cell imaging experiments to observe how cells adapt in real time to changes in substrate stiffness. Specifically, we tracked the nuclei, actin cytoskeleton, and focal adhesions of WI38 human fibroblasts grown on gels initially measured at the highest rigidity of this setting, 12 kPa (Movies S1 and S2 and Figure 2a).

As general control throughout these experiments and those presented below, we confirmed that cells seeded on standard tissue‐culture plastic or non‐degradable control PAA substrates and treated with 1 mm GSH displayed no significant changes in adhesion and mechanotransduction, as determined by paxillin staining patterns, cell/nuclear shape metrics, and YAP/TAZ localization when compared to untreated controls (Figure S3). Thus, responses observed on DPAA substrates arise solely from mechanical softening, rather than from any direct chemical effects of GSH on cells.

We started by capturing real‐time shifts in F‐actin structure during the softening process. For this, we used Fast‐Act, a live probe that allows natural actin assembly and disassembly. We made several interesting observations. At t_0_, the cells were stretched and showed abundant stress fibers aligned with their main axis, indicative of strong actomyosin contractility (Movie S1). After GSH pulse, we noticed a biphasic response in focal adhesions and peripheral stress fibers. During the first phase (t_0_–t_3_ of Figure 1e), corresponding to 0–60 min softening, and to substrate softening from 12 to 2.5 kPa, cells remain stretched.

To more precisely assess changes in cell‐substrate engagement, we monitored the dynamics of mature focal adhesions by using cells expressing Venus‐tagged Vinculin, a live cell fluorescent reporter for Vinculin, a marker of mature focal adhesions. This reagent thus identifies peripheral adhesions (namely corresponding to Focal adhesion‐stress fibers‐Focal adhesion triads, located at the cell outer border or sub‐cytoplasmic, those allowing cell spreading and attachment to the basal ECM), and subnuclear Focal adhesion (corresponding to Focal adhesion‐stress fiber‐LINC complexes, that primarily connect the basal side of the cell with the nuclear envelope [22]). Live imaging of Venus‐Vinculin expressing cells (Movie S2), revealed that in this early phase peripheral adhesions did not disassemble but rather remodel, gradually exhibiting a continuous, crawling‐like movement toward the cell center (their progressive repositioning can be followed over time in three colors in Figure 2a), but not decreasing in length and thickness. This suggests that pre‐formed peripheral focal adhesions are self‐organizing and self‐preserving.

In the second phase (t_3_–t_6_ of Figure 1e) of the softening response – during which substrate stiffness drops from 2.5 kPa to 1.2 kPa – peripheral adhesions suddenly and collectively disassemble (Figure 2b), remaining spatially confined to the outer cell border. This second step is accompanied by a dramatic cell rounding (Movie S1). To further delve into focal adhesions kinetics, we used immunofluorescence to monitor the clustering of the main RGD‐binding integrins, α_5_β_1_ and α_v_β_3_ (Figure 2c) at defined time points (t_0_–t_3_–t_6_). Similarly to what observed with Venus‐Vinculin, the density of these peripheral focal adhesions remained unchanged during the first phase of substrate softening and remained localized only at the outer cell border at the end of the second phase.

Different from peripheral adhesions, we could not avoid noticing that the subnuclear pool of adhesions (cyan color in Figure 2a) was much more labile and quantitatively snapped off just before t_3_ in our live experiments with Venus–Vinculin. We confirmed this result with timed immunofluorescence experiments using α_5_β_1_ and α_v_β_3_ and with an independent marker of focal adhesions, paxillin (Figure 2d and Figure S4).

Next, we focused our attention to the nucleus, as this is the stiffest organelle, and the nuclear envelope has been proposed to serve as mechanosensor, although the underlying mechanisms and their relevant kinetics remain incompletely understood [21]. We monitored nuclear projected area and nuclear shape during softening, as changes in nuclear elongation/flattening have been proposed to modulate nuclear pore mechanics and thereby influence YAP/TAZ nuclear entry [19]. For this, we performed a series of quantitative analyses comparing these parameters at time 0 (t_0_), at the end of the first and second phases of mechanosensing (i.e., at t_3_ and t_6_, respectively). We used DAPI to monitor nuclear shape and phalloidin staining to gauge the cell projected area. If nuclear shape‐dependent mechanisms contribute to the early mechanotransduction switch in this system, we would expect to detect rapid, temporally aligned changes in nuclear geometry during the same window. Up to the early phase (t_0_–t_3_), however, the nuclear shape and nuclear positioning did not show dramatic changes, with no changes in nuclear elongation and aspect ratio over the same timeframe (Figure 2e, middle and right panels). Conversely, nuclear shrinkage significantly occurs from t_3_–t_6_, suggesting that mechanical alterations of the nuclear envelope concur with loss of peripheral focal adhesion as dominant events of the second phase cellular response to substrate softening.

### 2.3 YAP/TAZ as Compass to Detect Early Responses to Cellular Mechanosignaling

Taken together, these observations raise an intriguing conundrum. We showed so far that several parameters that have been previously linked to mechanosensing in steady‐state conditions (i.e., peripheral focal adhesion, overall F‐actin organization, cell and nuclear shape) do not change in concert during a dynamic transition; instead, they are temporally separated and unfold with a reproducible order. This temporal separation prompts a key mechanistic question: does the sequence of structural changes reflect a hierarchy in mechanotransduction, and, if so, can it help distinguish initiating events from downstream, secondary adaptations? In addressing these questions, we chose YAP/TAZ as compass because its nuclear‐cytoplasmic redistribution marks the moment when mechanotransduction effectively reaches the nucleus and is translated into a nuclear transcriptional state. In other words, using YAP/TAZ timing as a nucleus‐level timestamp therefore provides a principled anchor to order upstream structural events during a continuous mechanical transition, distinguishing candidate initiating steps from later adaptations. We thus used YAP/TAZ nuclear vs cytoplasmic localization as detected by immunofluorescence as proxy that cells are in a mechano‐ON or ‐OFF state, respectively.

**FIGURE 3:**
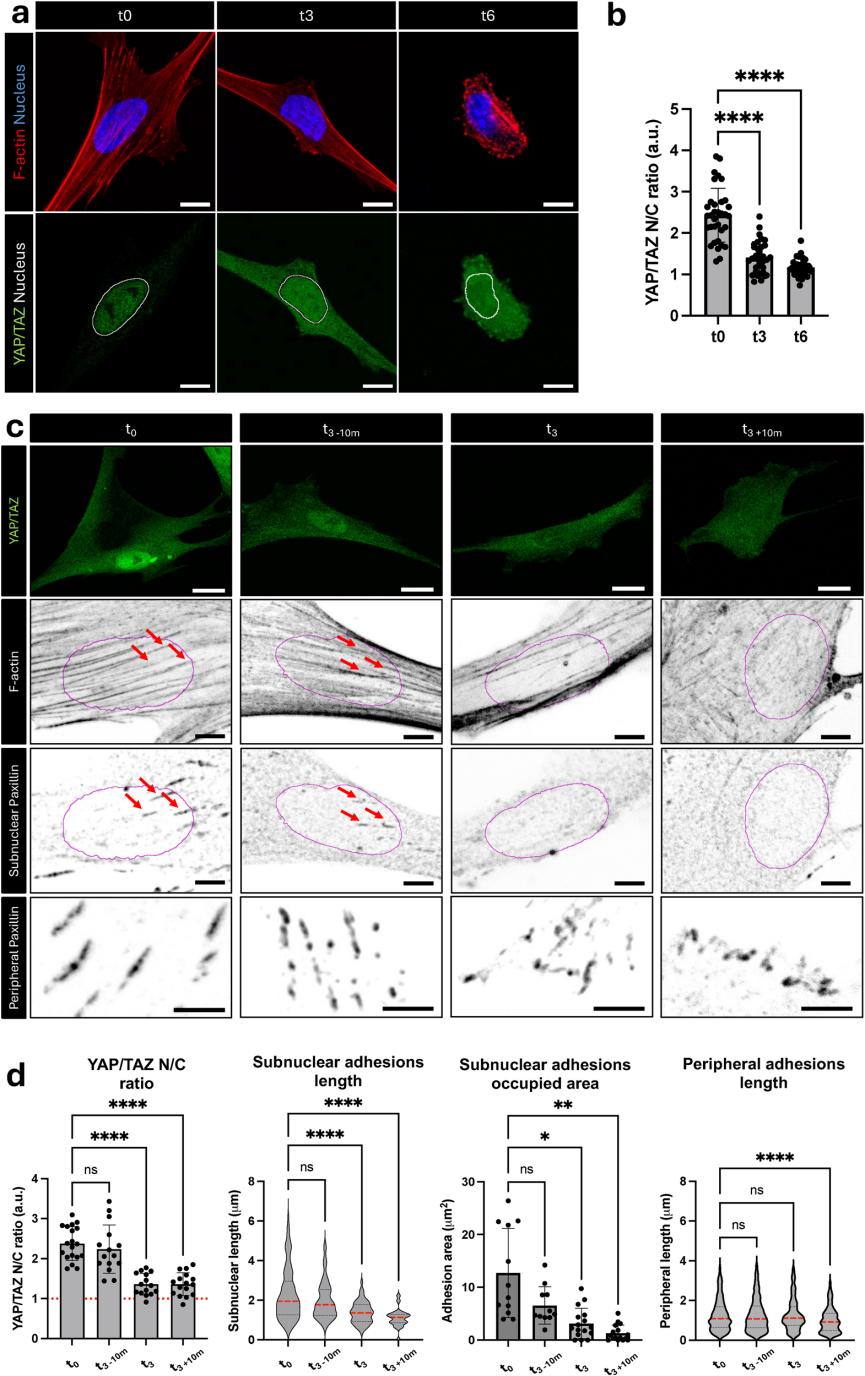
a) Representative immunofluorescence images of WI38 cells seeded on 12 kPa DPAA gel and fixed at different time points (t_0_, t_3_, t_6_) during gradual softening of dynamic gels. From the staining are visible: nuclei (in blue), YAP/TAZ (in green), and F‐actin (in red). F‐actin was stained with fluorescently labeled phalloidin to serve as cell shape reference. Scale bar 10 µm. b) Quantification of YAP/TAZ subcellular localization measured by the Nuclear to Cytoplasmic (N/C) ratio in WI38 cells seeded on DPAA gels with about 12 kPa of initial stiffness and fixed at different time points during gradual softening. *p*‐values t_0_–t_3_: <0.0001; t_0_–t_6_: <0.0001 (*n* = 34 for t_0_, *n* = 33 for t_3_, and *n* = 35 for t_6_). c) Representative immunofluorescence images showing the temporal evolution of YAP/TAZ localization, subnuclear F‐actin, paxillin‐ positive peripheral and subnuclear adhesions in short interval timepoints. Magenta circles indicate the outline of the nuclear projected area obtained by Hoechst counterstain. Scale bars 20 µm for YAP/TAZ, 5 µm for adhesions and F‐actin. d) Quantification of YAP/TAZ subnuclear localization, subnuclear and peripheral adhesions length and subnuclear adhesions occupied area. Statistical significance was evaluated with one‐way ANOVA with Welch correction. *p*‐values for YAP t_0_–t_3–10_: 0.9965; t_0_–t_3_: <0.0001; t_0_–t_3+10_:<0.0001, for subnuclear adhesions length t_0_–t_3–10_:0.3099; t_0_–t_3_:<0.0001; t_3_–t_3+10_:<0.0001 for peripheral adhesions length: t_0_–t_3–10_: 0.9999; t_0_–t_3_: 0.9998; t_0_–t_3+10_:<0.0001, for subnuclear adhesions area t_0_–t_3–10_: 0.1768; t_0_–t_3_: 0.0131; t_0_–t_3+10_: 0.0033. *n* = 19 for t_0_, *n* = 15 for t_3–10_, *n* = 15 for t_3_, *n* = 16 for t_3+10_.

We found that in cell exposed to our DPAA substrates, YAP/TAZ were nuclear at the initial stiffness of 12 kPa, as expected. By t_3_, however, cells already exhibited cytoplasmic YAP/TAZ enrichment (i.e., a mechano‐OFF state) as shown in Figure 3a,b. To further refine our observations, we directly quantified YAP localization and subnuclear vs peripheral adhesions in more time‐resolved window, assessing these values around t_3_ with ± 10’ resolution window in the very same cell. Strikingly, loss of YAP/TAZ nuclear localization occurs suddenly within 10 min preceding t_3_ (first row of Figure 3c,d); this timing coincides with diminished subnuclear stress fibers (Second row of Figure 3c), and disassembly of subnuclear adhesion (as marked by paxillin, third row of Figure 3c,d) while the peripheral adhesions remain unaffected (fourth row of Figure 3c,d). In other words, this early YAP/TAZ relocalization occurs within a timeframe that appears incoherent with events associated during changes in classic morphometric parameters of cellular mechanoresponse measured above – such as overall cell shape, nuclear major axis, or nuclear aspect ratio – that are instead later events (Figure 2e), and instead more closely associates to changes in subnuclear adhesions.

Finding that YAP/TAZ inactivation associates with the disappearance of subnuclear adhesions raised the possibility of a temporal connection between these events. Intriguingly, a recent study linked these structures to YAP/TAZ regulation in steady‐state mechano‐ON versus mechano‐OFF conditions [23]; yet, this work left unaddressed whether loss of subnuclear structures represents an initiating event during mechanical transitions or instead a consequence of broader cytoskeletal remodeling.

### 2.4 Nuclear Tethering Through Subnuclear Adhesions as Early Mechanosensor of ECM Softening

**FIGURE 4:**
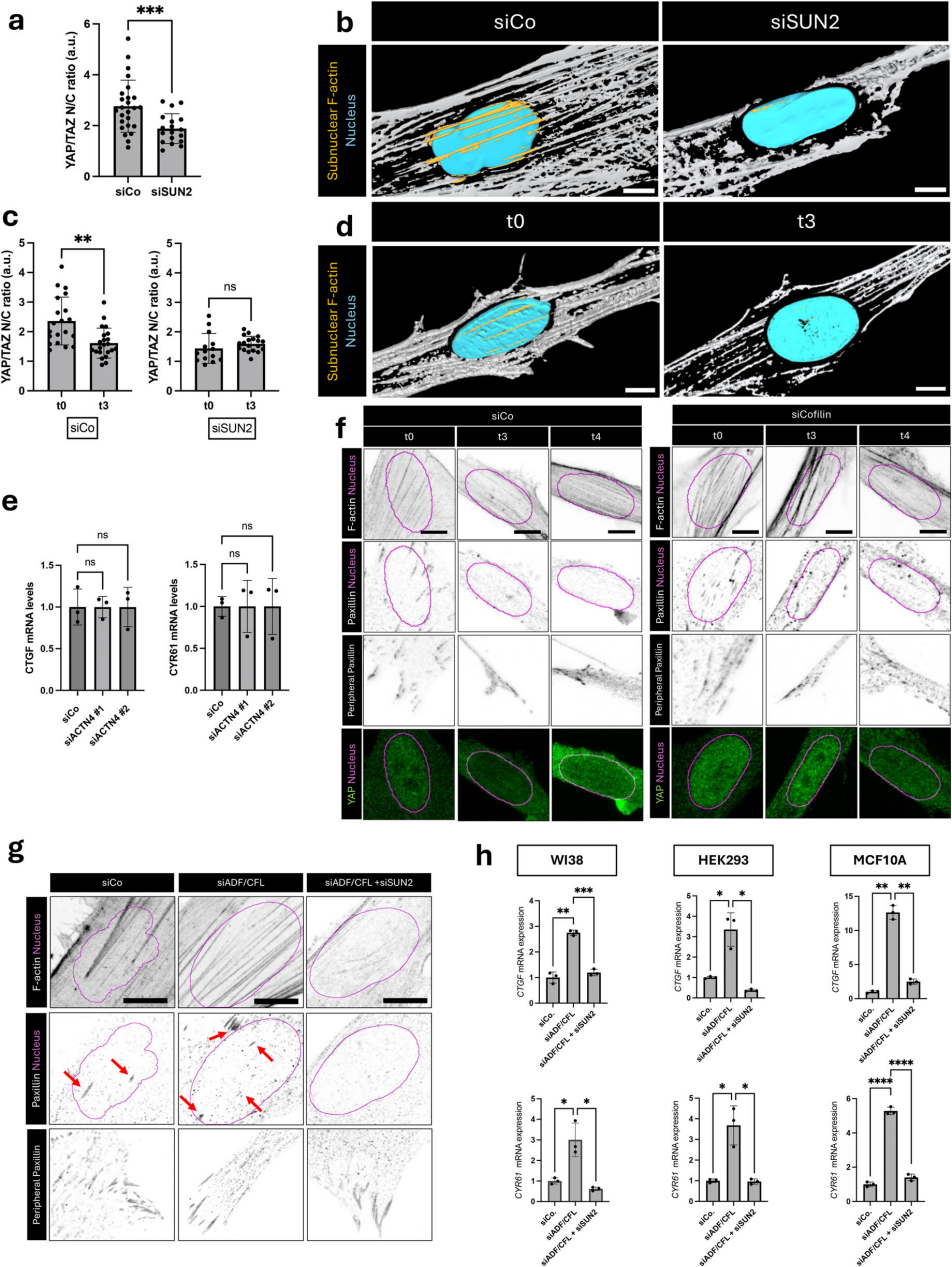
a) Quantification of YAP/TAZ Nuclear to Cytoplasmic (N/C) ratio in control vs SUN2‐depleted WI38 cells seeded on glass (*n* = 25 for siCo and *n* = 18 for siSUN2). Statistical significance was assessed with Student's *t*‐test with Welch's correction. *p*‐value = 0.001 b) Representative 3D IF reconstructions of control vs SUN2‐depleted WI38 cells seeded on glass. The nuclear lamina was labelled by laminA staining (LMNA, cyan) and F‐actin by phalloidin staining (F‐actin, grey). Subnuclear actin fibers are highlighted in orange in the 3D reconstruction (see Methods section). Scale bar 15 µm. c) Quantification of YAP/TAZ Nuclear to Cytoplasmic (N/C) ratio in control vs SUN2 depleted WI38 cells seeded on 12 kPa DPAA gels and fixed at different timepoints (t0, t3) of gel softening (control cells, *n* = 20 for t_0_ and *n* = 22 for t_3_, SUN2‐depleted cells, *n* = 15 for t0 and *n* = 19 for t3). Statistical significance was assessed with Student's *t*‐test with Welch's correction. *p*‐value = 0.0013 for siCo, *p* = 0.3108 for siSUN2. d) Representative 3D IF reconstructions of WI38 cells seeded on 12 kPa DPAA gels and fixed at different timepoints (t0, t3) during gradual softening. The nuclear lamina was labelled by laminA staining (LMNA, cyan) and F‐actin by phalloidin staining (F‐actin, grey). Subnuclear actin fibers are highlighted in orange in the 3D reconstruction (see methods). Scale bar 15 µm. e) qRT–PCR assessing the expression levels of the YAP/TAZ endogenous target genes CTGF and CYR61 in ACTN4‐depleted and control WI38 cells. *p*‐values were calculated with one‐way ANOVA test with Welch correction. *p*‐values. CTGF: siCO‐siACTN4#1 *p* > 0.9999, siCO‐siACTN4#2 *p* > 0.9999; CYR61: siCo‐siACTN4#1 *p* > 0.9999, siCo‐siACTN4#2 *p* > 0.9999. f) Representative immunofluorescence images of WI38 cells showing YAP/TAZ, basal actin and subnuclear adhesions at the indicated time points of gel softening. Magenta circles indicate the outline of the nuclear projected area obtained by Hoechst counterstain. Scale bars 10 µm. g) Representative IF images of WI38 cells treated with indicated siRNA showing subnuclear F‐actin, subnuclear and peripheral paxillin‐positive cell adhesions. Magenta circles indicate the outline of the nuclear projected area obtained by Hoechst counterstain. Red arrows highlight subnuclear adhesions. Scale bar 10 µm. h) qRT–PCR assessing the expression levels of the YAP/TAZ endogenous target genes CTGF and CYR61 in WI38, HEK293 and MCF10A cells treated with indicated siRNA. Statistical significance was evaluated with one‐way ANOVA with Welch correction. *p*‐values. WI38: CTGF siCo–siADF/CFL *p* = 0.0013 siADF/CFL–siADF/CFL + siSUN2 *p* = 0.0001; CYR61 siCo–siADF/CFL *p* = 0.0466 siADF/CFL–siADF/CFL + siSUN2 *p* = 0.0352; HEK293: CTGF siCo–siADF/CFL *p* = 0.0377 siADF/CFL–siADF/CFL + siSUN2 *p* = 0.0239; CYR61 siCo–siADF/CFL *p* = 0.0382 siADF/CFL–siADF/CFL + siSUN2 *p* = 0.0361; MCF10A: CTGF siCo–siADF/CFL *p* = 0.0020 siADF/CFL–siADF/CFL + siSUN2 *p* = 0.0012; CYR61 siCo–siADF/CFL *p* < 0.0001 siADF/CFL–siADF/CFL + siSUN2 *p* < 0.0001.

To elucidate the causal link between YAP/TAZ relocalization and subnuclear adhesion loss, we next investigated how perturbation of the LINC complex, linking the nuclear envelope to the actin cytoskeleton, interferes with subnuclear fibers and YAP/TAZ localization. In line with prior results in epithelial cells cultured on plastic substrates [23] siRNA‐mediated depletion of SUN2 in WI38 causes a selective loss of subnuclear actin coupled with a concomitant reduction of YAP/TAZ nuclear localization (Figure 4a,b). Importantly, SUN2‐depleted cells become insensitive to substrate softening, consistent with SUN2‐mediated nuclear tethering being necessary for the early YAP response to substrate softening (comparison between Figures 3b and 4c).

To directly visualize selective early loss of nuclear‐associated stress fibers during DPAA gel softening, we further implemented a machine‐learning‐based actin fiber classifier on 3D confocal reconstructions. This analysis revealed that nuclear‐associated stress fibers were preferentially lost at the early softening stage (t_3_), whereas peripheral fibers confirmed their distinct dynamics (Figure 4d). Notably, SUN2 depletion recapitulated this selective loss, reinforcing the link between nuclear tethering, subnuclear adhesion/stress fiber dynamics, and YAP mechanoregulation (Figure 4b,d). Complementarily, we also depleted α‐actinin4, that is enriched at peripheral adhesions and functionally essential for mechanosensing at the cell periphery in connection to ECM‐β_1_ integrin binding [24]. Interestingly, α‐actinin4 depletion does not significantly alter YAP/TAZ activity (assayed by their universal transcriptional readouts, CTGF and Cyr61; Figure 4e), in line with the view that peripheral adhesion remodeling is not the primary determinant of the early YAP/TAZ response in our system.

The results above suggest that the timing of YAP/TAZ nuclear exit occurs concomitantly to the loss of nuclear tethering to subnuclear stress fibers that are in turn anchored to subnuclear adhesive sites. If so, then increasing nuclear tethering by boosting stress fiber deposition should counter YAP/TAZ inhibition in our dynamic gels. To test this hypothesis, we seeded on DPAA WI38 cells depleted of F‐actin capping and severing factor Cofilin by transfection of validated siRNA [25] and followed their response to dynamic softening. Cofilin‐depleted cells not only showed the expected enhanced deposition of stress fibers [25], but changes their response threshold to substrate softening, sustaining and preserving subnuclear stress fibers and YAP/TAZ nuclear localization beyond t_3_ (Figure 4f and Figure S5a). Clearly, Cofilin depletion affects whole cell contractility by altering the stability/dynamics of all F‐actin fibers at steady state levels [25]. That said, by means of quantitative analyses of nuclear‐associated versus peripheral pools of F‐actin fibers, we found that during dynamic softening Cofilin depletion produces a disproportionately stronger preservation of the nuclear‐associated pool relative to peripheral one (Figure S5b). In other words, during the initial phase of ECM softening, peripheral adhesions and their associated F‐actin bundles remain largely uncompromised, such that even enhancing F‐actin polymerization has little effect on peripheral adhesion stability. By contrast, Cofilin depletion preferentially stabilizes the labile subnuclear F‐actin pool, supporting the notion that this pool is the dominant mechanosensitive actin population maintaining YAP/TAZ signaling under softening conditions.

Further supporting the role of subnuclear F‐actin as early YAP/TAZ mechanosensors, we pondered that a meaningful experiment would be one in which we selectively impair their anchorage to the nuclear envelope while concomitantly boosting overall stress fiber polymerization/tension. For this we combined ADF/Cofilin depletion with that of SUN2. We found that these cells retained peripheral actin/adhesion structures while losing subnuclear adhesion and subnuclear stress fibers (Figure 4g). Under these conditions, the cofilin‐driven increase in YAP/TAZ nuclear localization and transcriptional activities were suppressed (Figure 4h), indicating that the ability of increased actin bundling/contractility to sustain YAP depends on intact SUN2‐mediated nuclear coupling. We reproduced this result in WI38 fibroblasts and in epithelial MCF10A and HEK293 cells, supporting generality across cell types.

To link the above observations to YAP/TAZ‐dependent downstream effects, we considered that in stromal cells YAP/TAZ activation is required to blunt the cGAS/STING pathway [26]. We thus used cGAS activation as read‐out of YAP/TAZ inhibition in WI38 fibroblasts. This was monitored upon overexpression of dominant negative KASH, a tool that impairs tethering of the nuclear lamina through nesprin/SUN complexes to the actomyosin cytoskeleton. As shown in Figure S6, expression of dominant negative KASH induced perinuclear cGAS accumulation, as such confirming with an independent reagent that tethering of F‐actin to subnuclear adhesions is a primary driver of YAP/TAZ responses.

The tensional state of the nuclear envelope, mediated through its connection with subnuclear stress fibers, directly influences YAP/TAZ nuclear entry and activity. Importantly, Lamin A/C proteins are major determinants of nuclear stiffness and have been implicated in how nuclei adapt to mechanical stress [18, 21]. Perturbed nuclear envelope mechanics by depleting Lamin A, indeed impaired YAP/TAZ nuclear localization, recapitulating the phenotype observed upon SUN2 depletion and KASH‐DN overexpression (Figure S7).

### 2.5 A Mechanical “Ratchet Effect” to Dynamically Drive YAP/TAZ Activation

**FIGURE 5:**
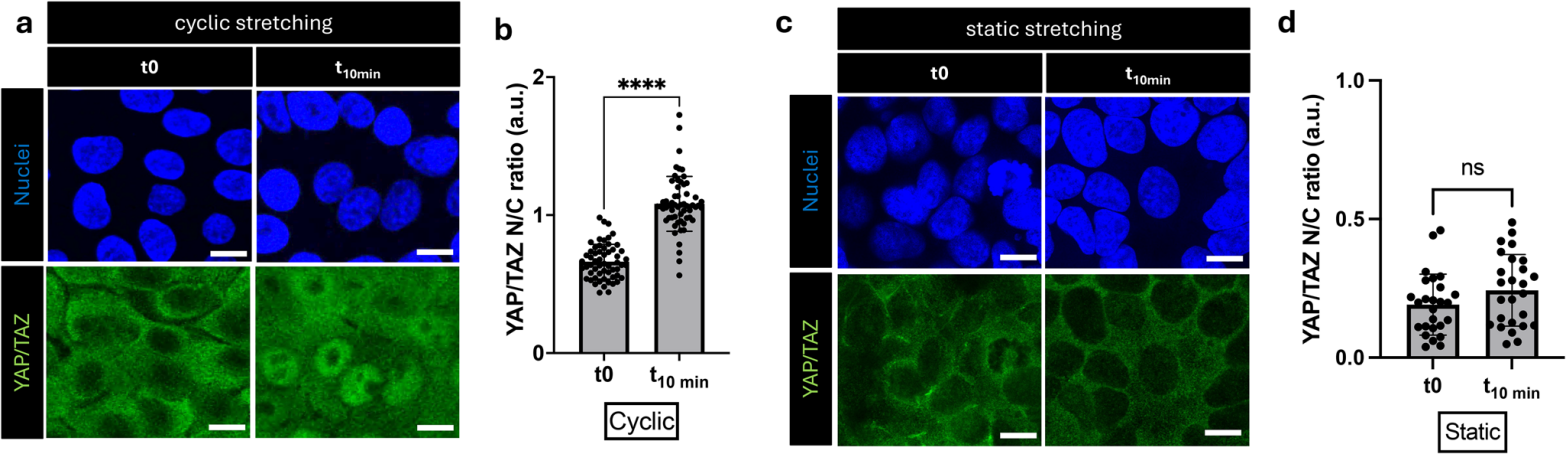
a) Representative images and quantification (b) of YAP/TAZ subcellular localization (*n* = 58 for t_0_ and t_10_) of MCF10A cells seeded in mechano‐OFF conditions (dense) before and after 10 min of cyclic stretching (1 Hz). Scale bar 10 µm. c) Representative images and quantification (d) of YAP/TAZ subcellular localization (*n* = 26 for t_0_ and t_10_) of MCF10A cells seeded in mechano‐OFF conditions (dense) before and after 10 min of static stretching. *p*‐values were calculated using the Welch *t*‐test. *p* < 0.0001 for cyclic stretching and *p* = 0.1259 for static stretching. Scale bar 10 µm.

The above results reveal a precise dynamic of events configuring a biphasic response to continuous substrate softening. Next, we asked whether the reverse transition, from a mechano‐OFF to a mechano‐ON state, follows similar rules. To test this, we applied in situ stretching to densely packed epithelial cell monolayers plated on a collagen I–functionalized PDMS membrane. Upon cyclic stretching (1 Hz), YAP/TAZ quantitatively relocalized from the cytoplasm to the nucleus within 10 min (Figure 5a,b). Strikingly, this did not occur when cells were subjected for the same duration to a constant (non‐cyclic) strain of comparable amplitude (Figure 5c,d). These results suggest that mechanoactivation can depend on temporal integration of repeated pull–relaxation cycles, rather than on the cumulative duration of strain per se. For simplicity, we refer to this behavior as “ratchet‐like,” in the sense that repeated cycles progressively drive the system toward a stable mechano‐ON state, whereas a sustained static input is less effective over the same time window. Intriguingly, temporal integration effects have also been connected to SUN2‐mediated nucleo–cytoskeletal coupling in other experimental contexts [27] (see Discussion).

### 2.6 Microtubule Restructuring as an Early, Time‐Aligned Layer During the YAP/TAZ Response

Next, we aimed to demonstrate that the precise timing of mechanosignaling is crucial to obtain direct evidence of the hierarchical unfolding of the leading events causing YAP/TAZ regulation. Microtubules have been recently reported to display dichotomic structural organization in unperturbed cells seeded on stiff vs. soft ECM substrates, as such matching comparable changes in F‐actin cytoskeleton in distinct mechanical states [28]. Moreover, microtubules’ centrosomal organization can sustain YAP/TAZ activation in epithelial cells by allowing retrograde transport and proteasomal degradation of AMOT, a potent YAP/TAZ cytoplasmic sequestering factor [23]. These findings were here replicated (Figure S8a–e). That said, from an epistemological perspective, the hierarchical connection and functional flow between the unfolding of these events still rest on inference: on the one hand, observations under static conditions cannot inform on the underlying set of events that brought a certain status quo; on the other, given that microtubules and F‐actin structures are so deeply interconnected, their functional perturbation alone cannot truly parse the temporal unfolding of natural events, risking to confound primary effects with their consequences, indirect compensations or long‐term adaptations.

**FIGURE 6:**
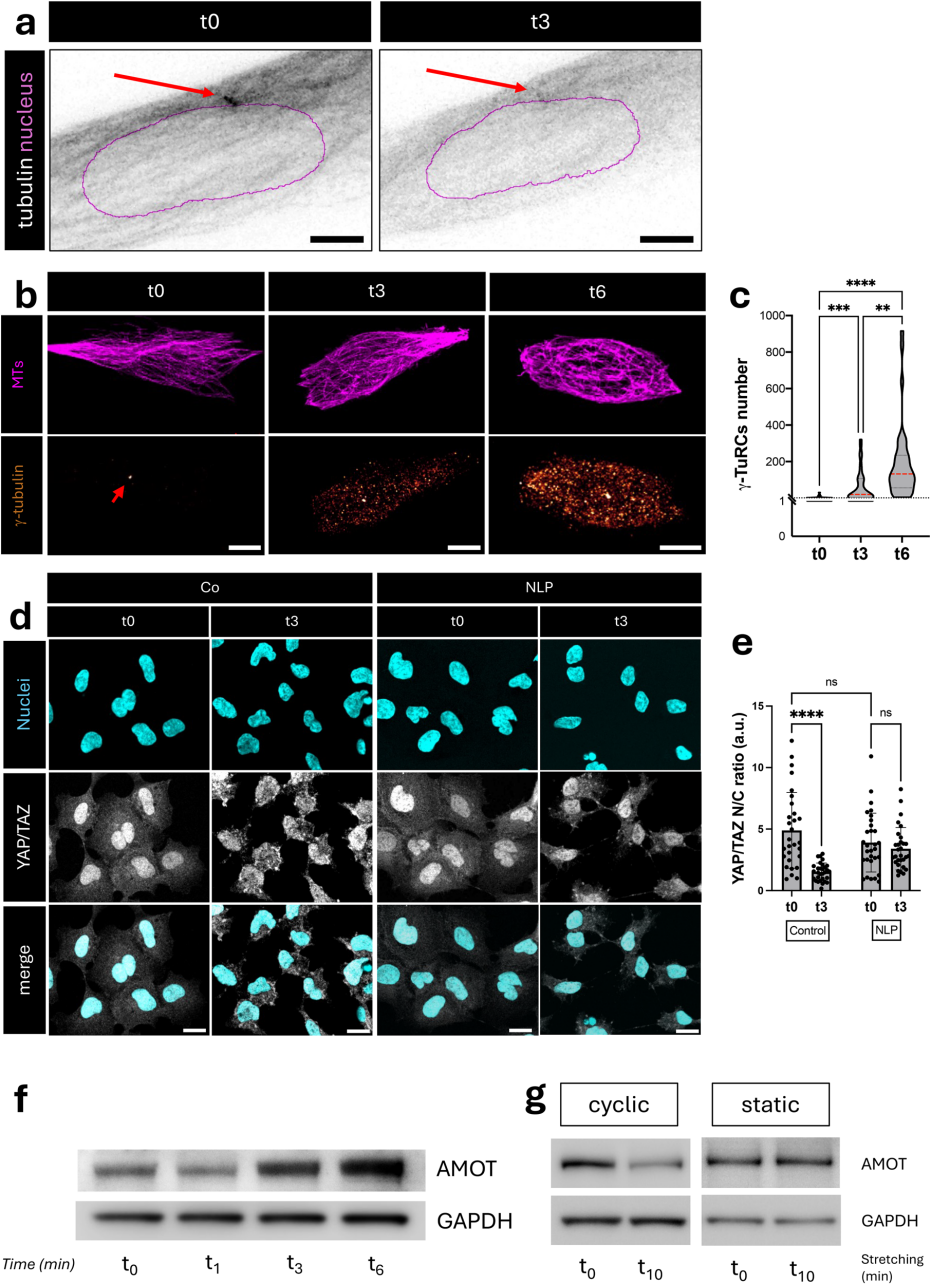
a) Still pictures taken during live imaging of nuclei and microtubules of WI38 labeled with Hoechst and SPY650‐tubulin respectively during gel softening (see Movie S3) showing MTOC disassembly around t3 (red harrow). Cells are seeded on gels with about 12 kPa of initial stiffness. Scale bar 10 µm. b) Representative IF images of WI38 cells seeded on DPAA hydrogels and fixed at different timepoints (t_0_, t_3_, t_6_) of gel softening showing MTs restructuring (obtained by α‐tubulin staining) and spatial distribution of γ‐tubulin–positive foci. Scale bar 10 µm. c) Quantification of γ‐TURCs (*n* = 38 for t_0_, *n* = 37 for t_3_ and t_6_) of WI38 cells seeded on 12 kPa DPAA gel at indicated time points during gradual softening of dynamic gels. Statistical significance was tested with one‐way ANOVA with Welch correction. *p*‐values. t_0_–t_3_: *p* = 0.0006; t_0_–t_6_: *p* < 0.0001; t_3_–t_6_: *p* = 0.0053. d) Representative IF images of YAP/TAZ in HEK293 cells transduced with empty or NLP1‐encoding lentiviruses and treated with cytochalasin D (Cyto.D, 1 µm, 1 h). Nuclei were counterstained with Hoechst. Scale bar 20 µm. e) Quantification of YAP/TAZ subcellular localization in HEK293 cells transduced with empty or NLP1‐encoding lentiviruses and treated as in (d). Statistical significance was tested with one‐way ANOVA with Welch's correction. *p*‐values *p* < 0.0001 for Control; *p* = 0.9255 for NLP; *p* = 0.6681 for t0 Control‐t0 NLP). f) Representative AMOT immunoblot of WI38 cells seeded on DPAA gels with about 12 kPa of initial stiffness at different time points during gradual softening. GAPDH serves as loading control. g) Representative AMOT immunoblot of HEK293 cells seeded in mechanically inhibiting conditions (dense) before and after 10 min of cyclic (left) or static (right) stretching. GAPDH serves as loading control.

Here, we thus set to follow the dynamic of microtubule restructuring within individual cells in our softening hydrogel platform. For this, we used SPY650‐Tubulin, a live‐cell probe that incorporates into the microtubule lattice, and complemented these observations with high‐resolution confocal images. At t_0_, microtubule architecture is dominated by a perinuclear microtubule‐organizing center (MTOC; arrow in Figure 6a and Movie S3), from which an asymmetrically oriented microtubule aster extends toward the cell periphery. During the early steps of mechano‐inhibition, we observed a rapid and reproducible transition from this centrosome‐centered perinuclear aster (mechano‐ON) to an alternative organization in which microtubules redistribute and progressively wrap around the nucleus, while nucleation becomes more dispersed. We quantified this remodeling by measuring the number and spatial distribution of γ‐tubulin–positive foci (γ‐TuRC‐associated nucleation sites), which increase and shift toward peripheral/cortical regions as softening proceeds (Figure 6b,c). Notably, these MT changes emerge within the same early time window in which we detect subnuclear stress fiber disassembly and YAP cytoplasmic relocalization in our dynamic assay.

To further probe the relationship between subnuclear actin‐based mechanosensing and MT organization, we phenocopied aspects of the softening response by time‐course inhibition of F‐actin with cytochalasin D (CytoD; dose titrated at 1 µm). Under these conditions, YAP/TAZ relocalized to the cytoplasm over ∼60 min. Strikingly, forcing retention of a centrosomal MT aster by overexpression of the centrosomal organizer protein NLP [29] rendered cells markedly less sensitive to CytoD and preserved nuclear YAP/TAZ throughout the experiment. Thus, MT organization is functionally required for YAP/TAZ mechanoregulation downstream of actin perturbation in this setting (Figure 6d,e), consistent with an MT‐dependent execution step in the pathway.

Previous work proposed AMOT turnover as a mechanorheostat: AMOT levels are low in mechano‐ON cells and high in mechano‐OFF cells, and AMOT regulation has been linked to actomyosin contractility, dynein‐dependent retrograde trafficking along a centrosome‐centered microtubule aster, and Hippo‐kinase inputs; crucially, AMOT loss‐of‐function impairs YAP/TAZ mechanomodulation [23]. However, are AMOT changes occurring fast enough to account for the initial YAP/TAZ switch during a mechanical transition, or is AMOT accumulation is instead a later consequence that reinforces cytoplasmic retention after YAP/TAZ have already relocalized?

We therefore quantified AMOT levels during DPAA gel softening. Remarkably, we found that AMOT accumulation becomes detectable at the early softening time point (t_3_) and continues to increase thereafter, matching the time window in which YAP/TAZ are turned off, subnuclear adhesions/nuclear‐associated actin disassemble, and microtubules remodel away from a centrosomal aster (Figure 6f). Together, these time‐resolved measurements move beyond prior static ON/OFF comparisons by placing AMOT regulation within the temporal sequence of events during an ON→OFF mechanical transition, consistent with a microtubule–AMOT layer contributing to rapid YAP/TAZ redistribution under dynamic softening.

We reported above that when mechanosignaling is turned ON from a starting mechano‐OFF state by applying a strain to the substrate, YAP/TAZ rapidly relocalize from the cytoplasm to the nucleus, but only under a cyclic stretching (see Figure 5a,b). This prompted us to ask whether AMOT turnover exhibits a similarly rapid correspondence during OFF→ON transitions. Strikingly, upon cyclic stretching (1 Hz), AMOT levels decreased markedly and became barely detectable after 10 min, i.e., within the same window in which YAP/TAZ accumulate in the nucleus (Figure 6g, left). Conversely, under static stretching conditions—where YAP/TAZ fail to accumulate in the nucleus—AMOT levels remained high (Figure 6g, right). Together, these data indicate that AMOT turnover tracks the cycle‐dependent mechanoactivation response and is compatible with the idea that rapid modulation of the AMOT cytoplasmic sink contributes to efficient YAP/TAZ nuclear accumulation during dynamic OFF→ON stimulation.

## 3 Discussion

Our work contains several elements of interest. The first is methodological. In order to address the spatiotemporal challenges of mechanotransduction, we established a dynamic polyacrylamide hydrogel platform that can be rapidly softened in situ and allows live imaging of cells during substrate softening. Our hydrogel formulation platform allows us to overcome a hitherto major limitation in mechanobiology research, namely, the inability to continuously modulate substrate stiffness while maintaining persistent cell‐substrate contacts. Indeed, traditional approaches often rely on abrupt switching – detaching cells and re‐plating them on substrates of different rigidities. As introduced, while several hydrogel platforms have been developed to allow post‐polymerization changes in stiffness – including light‐mediated [30], enzymatic [31], hydrolytic [32], or pH‐sensitive [33] systems – they all exhibit fundamental limitations for resolving mechanotransduction timing. Their stiffness changes occur either stepwise [30] or very slowly [32] (over hours or days), and often under conditions that preclude meaningful observation of fast cellular mechanosensing. Moreover, these platforms have largely been used to showcase material properties, not to address how mechanotransduction unfolds dynamically.

**FIGURE 7:**
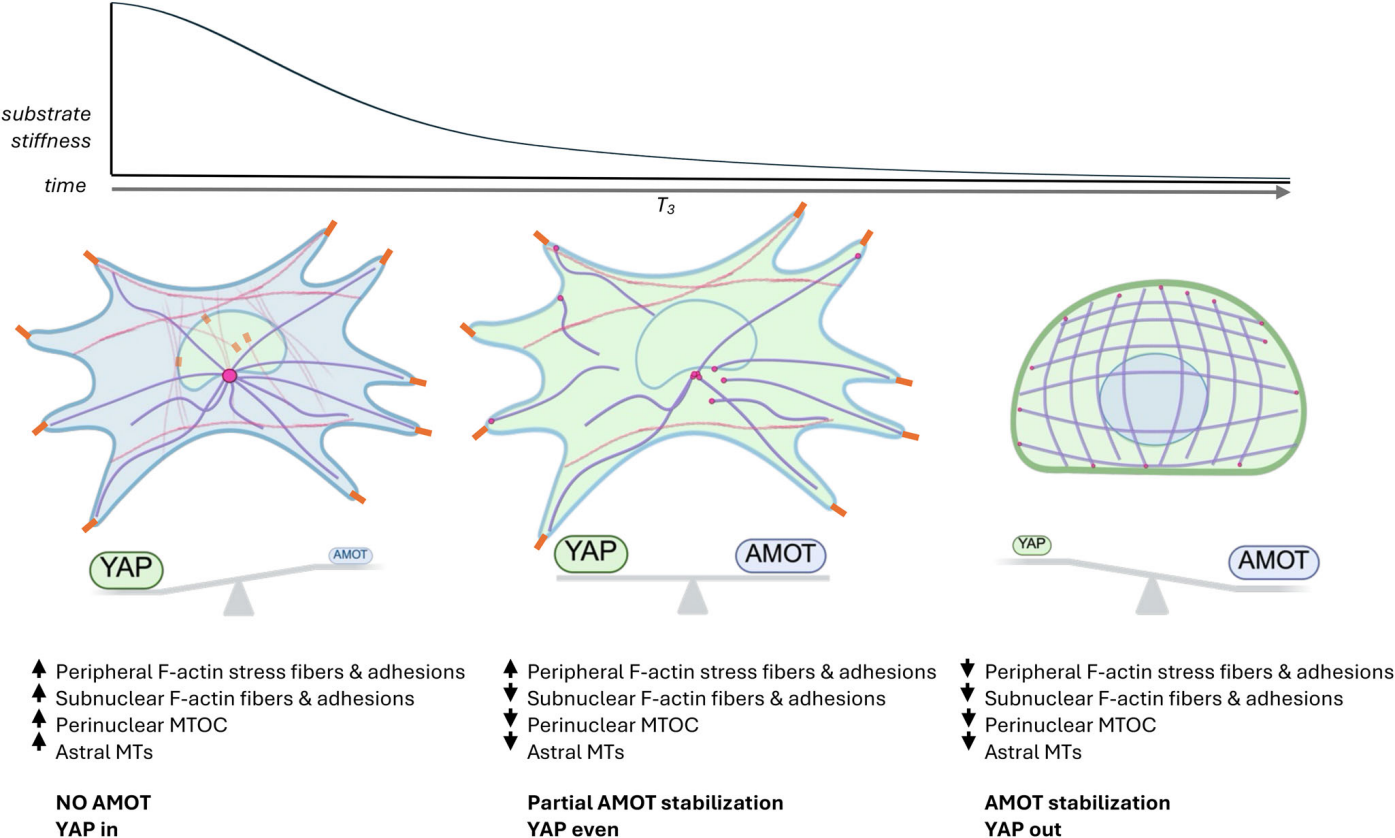
Schematic representation of the biphasic cellular mechanoresponse to substrate softening. The early phase of mechanosignaling, marked by cytosolic relocation of YAP/TAZ, is associated with initial subnuclear alterations in focal adhesions and actin fibers, together with temporally aligned microtubule reorganization and AMOT stabilization. Time‐resolved analyses indicate that YAP/TAZ inactivation coincides with the loss of these subnuclear adhesive structures, mediated by a microtubule–AMOT layer that promotes rapid cytosolic accumulation of YAP/TAZ. The later phase is characterized by nuclear shape remodeling, subsequent reorganization of peripheral adhesion architecture, and broader cytoskeletal remodeling. Collectively, these later events may stabilize the mechano‐OFF state and shape longer‐term transcriptional outcomes.

Leveraging our DPAA gel, here we advance on the timing by which mechanical information translates into nuclear events, an aspect of cell signaling that was so far enigmatic. Mechanotransduction, by its very nature, entails a myriad of interconnected events across different scales, from integrin clustering at focal adhesions to complex cytoskeletal reorganizations and nuclear envelope deformations [1, 3, 6]. Navigating through this complexity can be daunting, as, prior to this study, it was unclear whether all these molecular players act in concert during a dynamic mechanical transition or whether they instead relate to events unfolding with a deterministic order. Here, we fixed our compass on one dominant event of mechanotransduction: the nuclear accrual of YAP/TAZ, by which mechanics ultimately governs gene expression. By zeroing in on the timing of this fundamental signaling step, we were able to pinpoint the prime relationships and functional interdependencies between the earliest subcellular changes that trigger downstream transcriptional responses and YAP/TAZ activation. Equally important, we distinguished these early events from subsequent stages of cell reorganization that might be secondary, independent, or reinforcing the core nuclear mechanosignaling pathway (schematically summarized in Figure 7).

To start, addressing timing helps reconcile nuclear‐envelope‐based models of YAP/TAZ regulation with event timing [17, 19, 23, 27, 34, 35]. Changes in nuclear shape and envelope mechanics – including nuclear elongation/flattening and potential modulation of nuclear pore transport, as proposed in prior contributions [17, 19] – are likely to contribute to YAP/TAZ regulation. However, in our dynamic system these nuclear geometry changes emerge predominantly during the later phase of softening (t_3_→t_6_), after the initial YAP/TAZ nuclear exit and after loss of nuclear‐associated stress fibers/subnuclear adhesions. Nuclear pore permeability may matter mainly in the mechano‐ON state, where AMOT is typically low (for example, on stiff matrices that deplete AMOT). In contrast, pore‐centric models alone cannot readily explain regulation in the mechano‐OFF state: even on soft substrates, where pores are expected to be less permissive, loss of AMOT strongly increases YAP nuclear localization, and stabilizing AMOT in mechano‐ON cells can block YAP/TAZ nuclear entry despite permissive pores [23]. In light of these timing relationships, we favor sequential mechanistic layers operating under distinct mechanical regimes. In this view, early YAP/TAZ inhibition is triggered by rapid nucleo–cytoskeletal decoupling at the subnuclear adhesion/nuclear‐associated actin axis, in concert with a microtubule–AMOT layer that promotes cytoplasmic retention; subsequent nuclear‐shape remodeling, together with later changes in peripheral adhesion architecture and broader cytoskeletal reorganization, likely acts as a reinforcing/adaptive tier that stabilizes the mechano‐OFF state and shapes longer‐term transcriptional outputs. Consistent with this, YAP has been reported to be excluded from nuclei upon Lamin A/C depletion despite preservation of a smooth, distended nuclear envelope [17, 35], supporting the notion that a key determinant of YAP/TAZ nuclear localization is the integrity of the nucleo‐cytoskeletal continuum, rather than nuclear shape per se.

Our findings on the rapid dynamics of YAP/TAZ localization during in situ stiffness changes also align with evidence that cells on soft substrates display an acentrosomal microtubule organization that impairs AMOT proteasomal turnover, leading to AMOT accrual and formation of inhibitory YAP/TAZ–AMOT cytoplasmic complexes [23]. Importantly, our time‐resolved data indicate that AMOT does not rise gradually from the onset of softening: rather, AMOT accumulation becomes detectable at t3, coincident with YAP/TAZ cytoplasmic retention and disruption of the nuclear‐coupled adhesion/actin axis, and continues to increase thereafter. This timing highlights the existence of discrete mechanosensitive thresholds and suggests that YAP/TAZ shutoff is governed by early, time‐compatible control nodes rather than by slow, downstream remodeling alone.

These thresholds are unlikely to be absolute, as orthogonal inputs, most notably Hippo pathway activity, can modulate AMOT stability (e.g., via phosphorylation‐dependent escape from degradation) [23]. We therefore anticipate that Hippo‐inhibiting conditions, such as loss of cell–cell adhesion or specific GPCR cues [1], may shift the kinetics and/or threshold of YAP/TAZ inactivation in dynamic and physiologically‐mimicking conditions. Dissecting the contribution of LATS‐dependent mechanisms and related components will be an important direction for future work.

Finally, timing microtubule restructuring within single cells enables us to position prior steady‐state models – and their inferred hierarchies [23] – within a dynamic transition. We find that microtubules rapidly reorganize during the early softening window, transitioning from a centrosome‐centered perinuclear aster to a redistributed network with increased and more peripheral γ‐tubulin/γ‐TuRC nucleation sites and a nuclear‐wrapping architecture. Together, these observations place microtubule remodeling and AMOT availability within the same early window as the subnuclear adhesion/nuclear‐associated actin switch, and support the conclusion that AMOT regulation is temporally compatible with rapid YAP/TAZ responses in both directions of the mechanical transition (Figure 7).

It is also worth discussing that, leveraging our dynamic hydrogel platform, we could also carry out unprecedented live imaging experiments to monitor changes in F‐actin, microtubules, and Focal Adhesions organization during cellular adaptation to gradually decreasing substrate stiffness. Our observations revealed that preformed focal adhesions are simultaneously stable and highly dynamic: rather than collapsing in response to reduced force, these adhesions maintain cell‐substrate attachment by shifting centripetally, thereby continuously adapting to the decreasing rigidity of the substrate. This behavior marks, at least in part, a step forward in our understanding of stiffness sensing. The latter has been so far mainly conceptualized accordingly to the “molecular clutch” model, derived from static cellular studies in cells experiencing substrates of fixed degrees of stiffness [5]. According to the molecular clutch model, on softer ECM, substrate deformation absorbs part of the cell‐generated force, reducing the rate of force transmission from integrins to F‐actin. In this view, F‐actin engagement at focal adhesions becomes slower than the integrin‐ECM bond lifetime, ultimately leading to bond dissociation and focal adhesion collapse [36, 37]. Our dynamic, real‐time analyses of cells transitioning from stiff to softer substrates reveal a more nuanced scenario. During the initial phase of softening, peripheral focal adhesions do not abruptly collapse; instead, they remain largely intact while remodeling and crawling centripetally, preserving cell attachment, contractility, and overall cell shape. One plausible interpretation is that, once established on a rigid ECM, these adhesions remain engaged long enough to sustain continuous integrin–ligand turnover while the adhesion complex shifts rearward, effectively maintaining force transmission across a changing mechanical landscape. Strikingly, however, despite this remodeling resilience of peripheral adhesions, the behavior of subnuclear adhesions during declining (mechano‐ON→OFF) mechanical stimulation does instead appear more consistent with the classical clutch prediction, showing rapid disassembly as stiffness drops, in temporal proximity to YAP/TAZ nuclear exit.

Recent work on hydrogels undergoing repeated cycles of stiffening and softening further suggests that mechanosignaling is not always dictated by instantaneous force balance: cells can integrate transient mechano‐ON inputs over time and progressively increase focal adhesion activation and YAP nuclear accumulation [27, 34]. Inspired by these observations, we performed complementary experiments switching cells from a mechano‐OFF starting condition toward a mechano‐ON state by applying in situ stretching. Remarkably, within minutes, cyclic stretching induced robust mechanoactivation, as indicated by quantitative YAP nuclear accumulation together with degradation of its cytoplasmic sink AMOT. In contrast, when mechano‐OFF cells were exposed for the same duration to a constant (non‐cyclic) strain of comparable amplitude, they remained largely mechanically inhibited, failing to degrade AMOT and to accumulate nuclear YAP. These findings raise the possibility that the mechano OFF→ON transition may require repeated mechanical stimulation. While the molecular basis of such behavior remains to be defined in our system, it is compatible with emerging models in which focal adhesion reinforcement depends on time‐dependent maturation steps (including sequential mechanosensitive transitions within the integrin–talin–vinculin axis and cytoskeletal adaptation) that may require recovery periods between force applications [38]. Notably, temporal integration effects have been reported to depend on SUN2‐mediated nucleo‐cytoskeletal coupling [27], aligning with our evidence that nuclear anchorage to subnuclear adhesions constitutes a particularly sensitive sensor of mechanical state under dynamic conditions. All in all, the divergence between peripheral and subnuclear adhesion behaviors suggests that these adhesion systems may be functionally distinct in ways that remain largely unexplored.

We further note that mechanoactivation may exhibit temporal integration of pulsatile inputs – here termed “mechanical ratchet” conceptually analogous to classical models of cumulative morphogen sensing (e.g., originally by John Gurdon) [39]. Consistently, AMOT is rapidly depleted under cyclic (but not static) strain, paralleling fast YAP/TAZ nuclear accumulation. Conversely, during softening, YAP/TAZ switche off abruptly at a defined threshold, coincident with early AMOT accumulation. Together, these findings indicate that mechanotransduction is directionally asymmetric, with distinct kinetic constraints for OFF→ON versus ON→OFF transitions, underlining the need to focus on time‐resolved checkpoints.

In conclusion, the DPAA system provides a versatile platform for dissecting short‐lived, early mechanotransduction events that traditional approaches often overlook. By preserving continuous cell‐substrate interactions during substrate softening, we unveil a new dimension in the timing of mechanotransduction, suggesting that efforts to modulate subnuclear adhesion dynamics could be a potent strategy to control YAP/TAZ‐driven cellular processes in development, regeneration, and disease.

## 4 Materials and Methods

### 4.1 Non‐Adhesive Glass Preparation

Non‐adhesive hydrophobic glass slides were used as platform for the polymerization of DPAA hydrogels. A 1 mm thick glass substrate was first washed with water to remove any residual grease or powder from the surface. Then, each side of the slide was submerged in a 3N sodium hydroxide (NaOH) solution for 15 min to clean and prepare the glass slide for silanization. After repeated washing with water, the slide was left to dry in an oven at 140°C for about 40 min. After surface activation with a plasma cleaner (Harricks Expanded Plasma Cleaner), the glass slide was silanized by covering the surface with PlusOne Repel‐silane ES (Cytiva no. 17‐1332‐01). After 15 min, the slide was washed three times with pure ethanol and was left to dry in an oven at 80°C before use.

### 4.2 Synthesis of DPAA Hydrogels

To synthesize various formulations of dynamic polyacrylamide (DPAA) hydrogels, acrylamide (AA) solution (40 wt.% in water, Sigma–Aldrich no. 01697), bisacryloyl‐cystamine (BAC, Sigma–Aldrich no. A4929) solution (3 wt.% in AA 40 wt.%) and water were mixed to achieve the following compositions 5%AA 0.1%BAC or 7%AA 0.1%BAC or 5%AA 0.2%BAC or 5%AA 0.3%BAC or 7%AA 0.3%BAC. To introduce adhesive sites on the gel surface, the solution of AA and BAC was copolymerized with an acrylate‐PEG‐maleimide monomer (Lysan Bio, ACRL‐PEG‐MAL‐5000) previously conjugated with amino‐cysteine terminated synthetic peptide containing the adhesive sequence RGD, GRGDSPC. The conjugation reaction was carried out in water at room temperature: the RGD peptide and acrylate‐PEG‐maleimide were dissolved to a final molar ratio of 1:1 and mixed with the prepolymer solution at a fixed concentration of 3 mm for all formulations. Monomers solution was degassed for 15 min at 0.1 bar, to remove the dissolved oxygen which would inhibit the polymerization. Once degassed, the monomer solution was mixed with 1% v/v of 10 wt.% ammonium persulfate (APS, Sigma–Aldrich 09913) solution and 0.1% v/v of tetramethylethylenediamine (TEMED, Sigma–Aldrich T7024). Once mixed, the solution was poured inside proper PDMS molds positioned on non‐adhesive glass. Finally, an adhesive glass coverslip prepared by silanization of the surface with 3‐(Trimethoxysilyl)propyl methacrylate (TMSPM, Sigma–Aldrich 440159) is used to seal each PDMS mold and the reaction allowed to go to completion for 20 min. TMSPM functionalized glass provide a stable support for following cell seeding and analyses. Covalent anchoring and geometry of the gel strongly restrict its lateral swelling.

The PDMS molds and the glass coverslip dimensions are optimized according to the specific experiment: 250 µm thick PDMS ring and #1 18 mm round coverslips were used for fixed‐cell imaging, 50 µm thick PDMS stripes and #0 25 mm round coverslips were used to polymerize gels for live cell imaging.

Once polymerized, the gels were detached from the molds and placed in a petri dish submerged with MilliQ water. Water was then substituted with phosphate‐buffered saline (PBS 1X) and gels were left overnight to reach the swelling equilibrium before cell seeding.

Non‐degradable gels, used as a control for glutathione effect on cell behavior, were synthetized following the same procedure but substituting BAC with glutathione‐insensitive bisacrylamide (BA) crosslinker. Specifically, chemical composition was achieved mixing acrylamide solution (AA), 2% bisacrylamide solution (BA), and MilliQ water to reach the final composition: 7%AA 0.24%BA. RGD‐PEG conjugate was mixed as previously reported to reach a final concentration of 3 mm.

### 4.3 DPAA Softening

To induce controlled softening of DPAA hydrogels, glutathione (GSH, Sigma Aldrich no. PHR1359) was used as reducing agent. GSH was dissolved in Minimum Essential Medium (MEM, Thermo Fisher Scientific no. 31095029) supplemented with 10 wt.% fetal bovine serum (FBS, Thermo Fisher Scientific no. A5256701), 1 wt.% penicillin/streptomycin, and 1 wt.% glutamine to reach a final concentration of 1 mm. After complete dissolution, a proper volume of GSH solution was substituted to the culture medium to start degradation.

### 4.4 Hydrogels Mechanical Characterization

Micropipette aspiration was used to measure hydrogel stiffness. In brief, hydrogel samples were prepared attached to an adhesive glass coverslip and were left in 1× PBS overnight to reach the swelling equilibrium. For the measurement, a glass capillary connected to a syringe pump and a pressure sensor were mounted on top of an inverted microscope with the sample holder placed perpendicular to it. The gel was placed on the holder and the capillary was moved toward the surface of the gel to achieve a complete contact. A negative pressure was then applied to the sample surface through the capillary and was detected and registered using the pressure sensor. Simultaneously, an image of the gel aspirated length inside the glass pipette was taken with the inverted microscope. The analysis of the elastic modulus was done correlating the aspirated length and the pressure exerted with the Young modulus of the hydrogel through the model published previously [40].

The characterization of the degradation dynamics was performed preparing three samples for each timepoint. The samples were subjected to the glutathione solution and the degradation was stopped washing the samples for three times with 1X PBS. The mechanical properties were evaluated on two or three spots on the surface of each sample.

### 4.5 Lateral and z‐Swelling Measurement

To evaluate the hydrogel swelling along *x*,*y*, and *z*‐axis, fluorescent nanoparticles containing hydrogels were prepared and analyzed acquiring confocal images of the hydrogel surface. The image acquisition was performed with a Leica Stellaris 5 confocal microscope. Degradable hydrogels were positioned in an imaging petri dish and imaged with a 10X objective for 120 min after the addition of a 1 mm glutathione solution in culture medium. Hydrogel free surface was determined as the plane containing the last, on‐focus, set of fluorescent particles. Three images of the surface were acquired every 10 min. Lateral swelling was evaluated comparing the relative positions of arbitrarily selected particles of the surface. Z swelling was calculated as percentual swelling relative to the initial hydrogel thickness.

### 4.6 Cell Culture

WI38 cells were purchased from ATCC (CCL‐75). WI38 and transfected WI38 were cultured in Minimum Essential Medium (MEM) supplemented with 10 wt.% fetal bovine serum (FBS), 1 wt.% penicillin/streptomycin, and 1 wt.% glutamine. Cells were seeded on degradable hydrogels at a concentration of 2500 cells/cm^2^ for both fixed‐cells analysis and live cell imaging. Fixing or live imaging were performed 24 h after seeding. For in vitro vinculin overexpression studies in fibroblasts, WI38 cells were seeded at 30% confluency and transduced with virus diluted 1:4 v/v in culture medium in the presence of polybrene (Sigma) 8 µg/mL. For in vitro KASH‐DN and RFP‐cGAS overexpression in fibroblasts, WI38 cells were treated as in Sladitscheck et al. [26] Briefly, cells were brought to single‐cell suspension, overlaid with pTRIP‐CMV‐tagRFP‐FLAG‐cGAS (Addgene # 866756) and pCW57.1‐KASH‐DN [26] lentiviral supernatant and spun at 7.0 relative centrifugal force for 1.5 h; cells were then maintained with or without (as Control) 10 µg mL^−1^ doxycycline hyclate (Sigma–Aldrich) for 36 h.

MCF10A cells were a kind gift from F. Miller (Karmanos) and were cultured in DMEM/F12 (Gibco) with 5% horse serum, glutamine and antibiotics, freshly supplemented with insulin (Sigma–Aldrich), h‐EGF (Peprotech), hydrocortisone (Sigma–Aldrich) and cholera toxin (Sigma–Aldrich).

HEK293 cells were from ATCC and were cultured in DMEM (Gibco) supplemented with 10% FBS, glutamine and antibiotics.

NLP‐expressing HEK293 cells were obtained by transduction with plenti‐NLP‐c‐Myc‐DDK‐P2A‐puro (Origene #RC206094L3) followed by puromycin selection. NLP‐expressing cells were cultured as HEK293.

HEK293 AMOT tKO cells were obtained by CRISPR‐Cas‐mediated gene editing. Pre‐validated independent gRNAs targeting AMOT‐130 (sgAMOT130: GTAGAATTCACTGGAACTTG) AMOT‐L1 (sgAMOTL1: GGTGGAAATGAGAGGTTCCG), and AMOT‐L2 (sgAMOTL2: GTTACCTGACTTCAGCATGC) were obtained by oligo annealing and sequentially subcloned into pSpCas9(BB)‐2A‐Puro (PX459) ‐V2.0 (Addgene plasmid # 62988) to obtain a construct concomitantly expressing 3 sgRNAs (AMOT tKO‐A) from three independent U6 promoters. HEK293 cells were transfected with pSpCas9(BB)‐2A‐puro‐AMOTtKO‐A or pSpCas9(BB)‐2A‐puro‐sgGFP (GFP: GGAGCGCACCATCTTCTTCA) as negative control using TRANS‐IT LT1 transfection reagent following the manufacturer's instructions. Cells were selected by puromycin and independent clones were expanded. Selected AMOT tKO cells were cultured as HEK293.

For drug treatments, CytochalasinD was purchased from Sigma and used at a final concentration of 1 µm for 1 h, Nocodazole was purchased from Selleckhem and used at a final concentration of 5 µm for 1 h.

### 4.7 Live Imaging

Cells seeded on a degradable hydrogel specifically synthetized for live imaging and polymerized on thin #0 glass substrates (90–110 µm) were treated for 60 min with SPY650‐FastAct probe or SPY650‐tubulin (SpyroChrome) according to the manufacturer's instructions. Fifteen minutes before imaging, nuclei were counterstained with a Hoechst diluted solution (1:12500 v/v HOECHST 33342 solution in 1X PBS). Samples were washed twice with fresh medium, mounted on a metallic ring (Attofluor cell chamber) and filled with 400 µL of probe containing‐culture medium. Live imaging was performed on a Leica Stellaris 5 confocal microscope equipped with Okolab cage incubator and a 63X oil immersion objective. Live data mode was used to establish an automated imaging protocol consisting of three steps iteratively repeated until completion of the imaging. A first step consisting of an auto‐focus required to take into account the z‐shifting of the gel surface during degradation is followed by a z‐stack imaging of the cell. A 20 µm stack is acquired in 26 planes (every 0.8 µm). The third and final step is a pause step of 30 s.

The hydrogel softening is initiated substituting culture medium with a 1mm solution of glutathione in fresh medium. Raw files were then analyzed with Fiji software.

### 4.8 RNA Interference

siRNA transfection was performed with Lipofectamine RNAi‐MAX (Thermo Fisher Scientific) in antibiotic‐free medium according to the manufacturer's instructions. Transfected cells were seeded on degradable hydrogels 24 h post siRNA transfection. For dual siRNA transfections, transfections were performed 8 h apart. Cells were harvested 48–72 h post siRNA transfection. Used human siRNAs are listed below:

CFL: GGAGGAUCUGGUGUUUAUC

ADF: GCUUUGUAUGAUGCAAGCUUU

SUN2: CACCCGAUGUUCUGAGACCUA

ACTN4#1: CAGGACAUGUUCAUCGUCCAU

ACTN4#2: UCCACUCUGUAUCUAUGCAAA

LMNA: equimolar mix of four independent pre‐validated siRNAs: 1) CCAGGAGCTTCTGGACATCAA; 2) AACTGGACTTCCAGAAGAACA; 3) CCCACCAAAGTTCACCCTGAA; 4) CAGGCAGTCTGCTGAGAGGAA

siControl was purchased from Qiagen (ref. code 1027280)

### 4.9 Lentivirus Production

The pLL3.7‐CMV‐Vinculin‐Venus vector was generated by subcloning Vinculin‐Venus (Addgene #27300) into the pLL3.7‐CMV vector (Addgene #11795) through enzymatic digestion. For lentiviral particles production, HEK293T were seeded at 40% confluency in 10 cm dish (Corning) and transfected with 10 µg pLL3.7‐CMV‐Vinculin‐Venus, 2.5 µg pMD2.G (Addgene #12259) and 7.5 µg pPAX2 (Addgene#12260) by using Trans‐IT X2 Dynamic Delivery system (Mirus Bio) according to manufacturer's instructions. Lentivirus particles were recovered 48 h post‐transfection by filtering HEK293T supernatant through 0.45 µm filter (Sarstedt).

### 4.10 Static Hydrogel Fabrication

Fibronectin‐coated hydrogels were obtained as previously described [17] with minor modifications. Hydrogel formulations in AA (Acrylamide), BA (bis‐acrilamide), TEMED (tetramethylethylenediamine), and APS (ammonium persulfate) were as follows: 3.5% w./v. AA, 0.03% w./v. BA, 1:1000 v./v. TEMED and 0.1% w./v. APS (0.3 kPa); 3% w./v. AA, 0.15% w./v. BA, 1:1000 v./v. TEMED and 0.1% w./v. APS (3 kPa); and 8% w./v. AA, 0.48% w./v. BA, 1:1000 v./v. TEMED and 0.2% w./v. APS (40 kPa). In all the compositions, AA% represents the sum of acrylamide and N‐hydroxyethyl acrylamide, which is fixed at 0.1 M. Cells (4 × 10^4^ cells per cm^2^) were seeded in a drop of complete culture medium on top of fibronectin‐coated hydrogels; Cells were analyzed 24 h after seeding.

### 4.11 Immunofluorescence

Immunofluorescence was performed on 4% PFA‐fixed cells using anti‐YAP/TAZ (Santa Cruz Biotechnology no. sc‐101199 1:100), anti‐vinculin (Invitrogen no. MA5‐11690 1:100), anti‐paxillin (abcam no. ab32084 1:50), anti‐α5β1 (Novusbio no. NBP‐252680 1:100), anti‐αvβ3 (Sigma–Aldrich MAB1976 1:100) and anti‐LMNA/C (Santa Cruz Biotechnology no. sc‐376248, 1:200) as primary antibodies. F‐actin was stained with Alexa Fluor 568 Phalloidin (Thermo Fisher Scientific no. A12380 1:100). Goat anti‐mouse IgG1 Alexa fluor 647 (Thermo Fisher Scientific no. A21240 1:100) Goat anti mouse IgG2a Alexa fluor 488 (Thermo Fisher Scientific no. A21131 1:100), Goat anti‐rabbit Alexa fluor 647 (Thermo Fisher Scientific no. A21245 1:100) were used as secondary antibodies. For microtubules immunofluorescence, cells were fixed and permeabilized with a solution of 3% v/v PFA, 0.2% Glutaraldehyde, 0.25% triton X100 in PBS for 15 min as previously reported [41]. Samples were washed twice in PBS and residual aldehydes were reduced to alcohol incubating for 15 min on ice with a solution of sodium borohydride (1 mg/mL) in a quenching buffer (10 mm MES, 150 mm NaCl, 5 mm EGTA, 5 mm MgCl_2_, 5 mm glucose; pH 6.1). Microtubules were stained with anti‐α‐tubulin (abcam no. ab18251 1:500) and anti‐γ‐tubulin (Santa Cruz Biotechnology no. sc‐17787 1:500) as primary antibodies and Goat anti‐rabbit Alexa fluor 568 (Invitrogen A11011 1:200) and Goat anti‐mouse Alexa fluor 488 (Invitrogen A11029 1:200) as secondary antibodies. All samples were counterstained with Hoechst 33342 dye (Thermo Fisher Scientific no. 62249 1:1000) to label cell nuclei, unless differently specified. Confocal images were acquired with a Leica Stellaris 5 and analyzed using Fiji.

The YAP/TAZ nuclear to cytosolic ratio was calculated creating a pipeline in CellProfiler software. The CellProfiler sequence was built to obtain a mask of the Nuclear Projected Area (NPA) analyzing the nuclear signal, a mask of the cellular shape based on phalloidin signal, and a mask of the cytosolic area obtained subtracting the NPA to the cell projected area. The Nuclear to Cytoplasmic ratio (N/C) was then calculated by the software on the YAP/TAZ channel as the ratio between the mean signal intensity on the nuclear mask and the mean signal intensity on the cytosolic area.

For adhesion measurements, images were processed applying a background subtraction process with a 30‐pixels rolling ball radius and a top hat filter with a ten‐pixels radius. Adhesions length was evaluated building a custom Cellprofiler pipeline. This includes a non‐local means noise reduction, a thresholding step and a segmentation step based on watershed algorithm. The pipeline automatically calculates the major and minor axis of the detected objects. A minimum of three different ROIs for each cell has been analyzed for the peripheral adhesions while the area described by the nuclear outline was used as a mask for the analysis of the subnuclear adhesions.

For subnuclear to total F‐actin ratio quantification, ROIs of the nuclear projected area and cell shape were obtained by Hoechst and fluorescent phalloidin signals respectively. Subnuclear F‐actin and total F‐actin signals were measured using Fiji‐ImageJ analysis software.

For reconstruction of subnuclear F‐actin fibers we used AIVIA (Leica) software as previously described [23]. Briefly, a machine‐learning pixel classifier algorithm was trained and applied to highly resolved confocal images to differentially segment F‐actin fibers in direct contact with the nuclear lamina.

For γ‐TURCs quantification, the number of γ‐Tubulin‐positive dots per cell was counted by using Fiji‐ImageJ analysis software.

### 4.12 Western Blots

Immunoblots were carried out as previously described [42]. Briefly, samples were run in 4%–12% SDS‐PAGE in denaturing conditions. Proteins were transferred on PVDF membranes and incubated with the following antibodies diluted 1:1000 in 0.5% milk in TBS‐Tween20 0.05% (TBSt): anti‐AMOTL1 (Sigma, HPA001196), anti‐GAPDH (Millipore, MAB374). Incubation of primary antibodies was performed at 4°C overnight. After washes in TBS‐Tween, incubation with HRP‐conjugated secondary antibodies was performed for 1 h at room temperature. Membranes were washed in TBS‐Tween as before and a 1:1 v/v solution of SuperSignal West PicoPLUS Stable Peroxide: Luminol/Enhancer (Thermoscientific) was used as substrate for the chemiluminescence reaction. Images were acquired using ImageQuant800 machine and analyzed with Fiji‐ImageJ software.

### 4.13 Cell Stretching

Cell stretching experiments were performed on a MechanoCulture B1 stretching device (CellScale). HEK293 and MCF10A were seeded at high confluence on 55 mm PDMS membranes previously coated with a 10 µg/mL solution of collagen I. The covalent coating was achieved washing the membrane with isopropanol and, once dried, activating the surface with an air plasma treatment. Subsequently, activated PDMS was submerged in a 10% v/v solution of APTES (3‐Aminopropyl)triethoxysilane in ethanol for 2 min to introduce amines on the surface. Covalent conjugation with Collagen I was obtained immersing the silanized membranes in a 3% v/v glutaraldehyde solution for 20 min and, after two washes with PBS, incubating the membrane with a diluted collagen solution for 1 h at 37°C.

Cell stretching experiment were performed 24 h after cell seeding, by mounting the membranes on the stretching device. Cyclic regime was obtained imposing a stretching protocol composed of three phases: a stretch phase of 1 s, a hold phase of 1 s, a recover phase of 1 s (maximum stretch 5.5 mm) and a stretching frequency of 1 Hz. Static regime was performed keeping the membrane stretched for 600 s at the maximum stretch applied for the cyclic regime. Once treated, membranes were cut into two halves to perform immunofluorescence and western blot on cells.

### 4.14 Statistical Analysis

Values reported in Figures are means and standard deviations. Student's *t*‐test with Welch's correction and one‐way ANOVA with Tukey's multiple comparison and with Welch correction were performed as indicated in figure captions using GraphPad Prism 9 and considering a confidence interval of 95%. Data were tested for outliers through the ROUT method with Q = 1% where indicated. Data reported are representative of those obtained with three independent replicates.

## Author Contributions

**Alessandro Gandin**: Conceptualization: Lead; Data curation: Lead; Formal analysis: Lead; Investigation: Lead; Methodology: Lead; Validation: Lead; Writing – original draft: Lead; Writing – review & editing: Equal. **Giada Vanni**: Data curation: Lead; Formal analysis: Lead; Investigation: Lead; Methodology: Lead; Visualization: Equal; Writing – review & editing: Equal. **Veronica Torresan**: Investigation: Supporting; Methodology: Equal; Validation: Supporting. **Margherita Pelosin**: Investigation: Supporting; Methodology: Equal. **Rebecca Busetto**: Investigation: Equal; Methodology: Equal. **Anna Citron**: Investigation: Supporting; Methodology: Supporting. **Ambela Suli**: Methodology: Lead. **Paolo Contessotto**: Investigation: Supporting; Methodology: Supporting; Validation: Equal. **Carlo Jr Albanese**: Investigation: Equal; Methodology: Equal. **Francesca Zanconato**: Methodology: Lead; Supervision: Equal; Validation: Supporting. **Tito Panciera**: Conceptualization: Lead; Data curation: Lead; Formal analysis: Lead; Supervision: Lead; Writing – original draft: Lead; Writing – review & editing: Lead. **Stefano Piccolo**: Conceptualization: Lead; Data curation: Lead; Funding acquisition: Lead; Supervision: Lead; Writing – original draft: Lead; Writing – review & editing: Lead. **Giovanna Brusatin**: Conceptualization: Lead; Data curation: Lead; Funding acquisition: Equal; Supervision: Lead; Writing – original draft: Lead; Writing – review & editing: Lead.

## Funding

This research has received funding from the following agencies/charities: FONDAZIONE AIRC under 5 per Mille 2019 – ID. 22759 program, and under IG 2019 – ID. 23307 project to S.P.; the European Research Council Executive Agency (ERCEA) under the ERC‐2022‐ADG Grant Agreement n. 101098074‐CHARTAGING to S.P.; STARS@UNIPD, “GF‐MET‐Growth factor signaling in metastatic organotropism” to F.Z; and the National Center for Gene Therapy and Drugs Based on RNA Technology, funded in the framework of the National Recovery and Resilience Plan (NRRP), Mission 4 “Education and Research”, Component 2 “From Research to Business”, Investment 1.4 “Strengthening research structures for supporting the creation of National Centres, national R&D leaders on some Key Enabling Technologies”, funded by the European Union ‐ Next Generation EU, Project CN00000041, CUP C93C22002780006, Spoke n.2 (“Cancer”) to S.P., and Spoke n.5 (“Inflammatory and Infectious Diseases”) to G.B.; and Fondazione Cariverona through the project “Ricerca e Sviluppo 2022” number 52322 to G.B.

## Conflicts of Interest

The authors declare no conflicts of interest.

## Supporting information




**Supporting File 1**: advs74751‐sup‐0001‐SuppMat.docx.

**Supporting File 2**: advs74751‐sup‐0002‐Movie S1.mp4.

**Supporting File 3**: advs74751‐sup‐0003‐Movie S2.mp4.

**Supporting File 4**: advs74751‐sup‐0004‐Movie S3.mp4.

## Data Availability

The data that support the findings of this study are available from the corresponding author upon reasonable request.
