# Supplementary material for: Timing Mechanotransduction: Mechanically Dynamic Biomaterials Reveal the Temporal Hierarchy of YAP/TAZ Control Nodes
Source: Adv Sci (Weinh). 2026 Mar 19;13(30):e15210. doi: 10.1002/advs.202515210 (PMC13248771; doi:10.1002/advs.202515210)
Supplement: Supplementary file 1 — Supporting File 1: advs74751‐sup‐0001‐SuppMat.docx. [file ADVS-13-e15210-s001.docx]

**SUPPLEMENTARY INFORMATION**

**Timing mechanotransduction: mechanically dynamic biomaterials reveal the temporal hierarchy of YAP/TAZ control nodes**

Alessandro Gandin^1,2*^, Giada Vanni^3*^, Veronica Torresan^1,2^, Margherita Pelosin^1,2^, Rebecca Busetto^1^, Anna Citron^3^, Ambela Suli^3^, Paolo Contessotto^3^, Carlo Albanese^3^, Francesca Zanconato^3^, Tito Panciera^3$^, Stefano Piccolo^3,4,$^ and Giovanna Brusatin^1,2,$^

^1^Department of Industrial Engineering, University of Padova, Padova, 35131, Italy

^2^INSTM Padova RU, University of Padova, Padova, 35131, Italy

^3^Department of Molecular Medicine, University of Padua School of Medicine, Padua, Italy

^4^IFOM, The FIRC Institute of Molecular Oncology, Padua, Italy

^*^ These authors equally contributed to the work

^$^ Co-corresponding authors

**Table S1:** Chemical compounds used for the hydrogel synthesis and functionalization.

**Table S2:** Composition of different DPAA formulations prepared with 3mM of RGD. The initial modulus (E_in_, measured after 1 day swelling) and the final modulus (E_1h_, measured after 1h degradation, with 1mM GSH in PBS solution) are measured by micropipette aspiration (columns 3 and 4 respectively).

**Table S3:** Elastic modulus of degradable gel used for the seeding experiments (7%AA, 0.3%BAC, 3 mM of RGD), after different degradation times with GSH 0.5 or 1mM. The moduli are measured by the micropipette aspiration method.


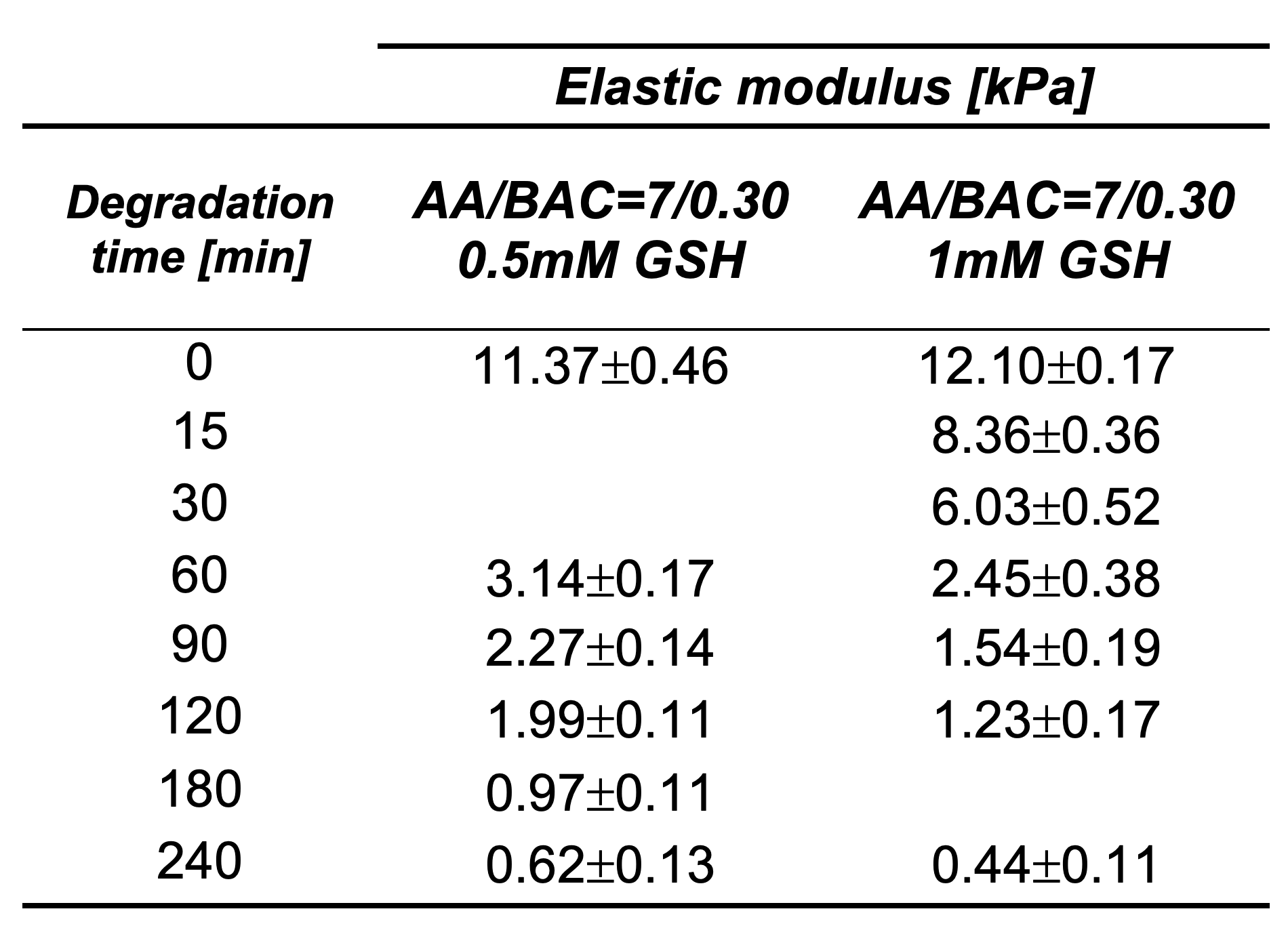


**FIGURE S1**

**Figure S1**: Bulk stiffnesses measured at different time points during gel softening with an initial stiffness of about 12kPa with 0.5 or 1mM GSH (red and blue line, respectively) for 4h. The degradation has been followed until a plateau value of stiffness is reached.

**FIGURE S2**

**Figure S2**: Swelling behavior along the z axis (thickness) measured during gel softening (GSH 1 mM). Measurements were acquired recording the position of the gel surface through confocal imaging of fluorescent nanoparticles embedded in the hydrogel. Data are reported as mean and standard deviations of swelling values measured in 3 different positions of the gel.

**FIGURE S3**

**Figure S3**: Representative immunofluorescence images of WI38 cells seeded on non-degradable PAA hydrogels. Scale bar 20 μm. Quantifications of YAP/TAZ localization and cells shape parameters of WI38 cells seeded on glass and non-degradable PAA hydrogels (glass: n=20 for untreated, n=42 for GSH-treated; non-degradable gels: n=16 for untreated, n=19 for GSH-treated). Cells were treated with 1 mM GSH. Statistical significance was tested with Welch t-test. P values. Glass: p=0.6835 for nuclear major axis, p=0.8859 for cell major axis, p=0.2067 for YAP/TAZ N/C ratio. Non-degradable gels: p= 0.5651 for nuclear major axis, p=0.8663 for cell major axis, p=0.5940 for YAP/TAZ N/C ratio

**FIGURE S4**

**Figure S4**: Representative immunofluorescence images of WI38 cells seeded on DPAA gel with an initial stiffness of about 12 kPa and fixed at the indicated timepoints during gel softening showing subnuclear adhesions (α_5_β_1_, α_v_β_3_ or paxillin) disassembly at t_3_. Magenta circles represent the outlines of the nuclear projected area. Red arrows highlight the main subnuclear adhesions. Scale bar 5 μm

**FIGURE S5**

**Figure S5:** Quantification of YAP/TAZ subcellular localization in Cofilin depleted and control WI38 cells (siCo: n=7 for t_0_, n=6 for t_3_, n=7 for t_4_. siCofilin: n=7 for t_0_, n=6 for t_3_, n=10 for t_4_) seeded on 12kPa DPAA gel and fixed at different timepoints during gradual softening. Statistical significance is evaluated with one way ANOVA with Welch correction (p values: siCofilin t_0_-t_3_: 0.1224; t0-t4: 0.0010; siCo t_0_-t_3_: 0.0272; t0-t4: 0.0303). c) Subnuclear to peripheral actin fibers quantifications of control and Cofilin depleted WI38 cells seeded on 12kPa DPAA gel and fixed at different timepoints during gradual softening. p values SiCo p=0.0342, siCofilin p=0.7480 (siCo: n=6 for t_0_, n=5 for t_3_; siCofilin: n=7 for t_0_, n=5 for t_3_)

**FIGURE S6**

**
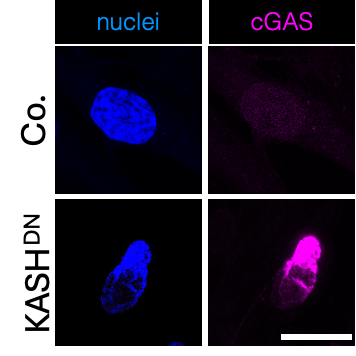
**

**Figure S6:**  Representative immunofluorescence images showing perinuclear accrual of cGAS (RFP-cGAS) in control (Co.) or dominant-negative KASH (KASH^DN^) expressing WI38 (TetO-EGFP-KASH^DN^). Scale bar 10 μm.

**FIGURE S7**

**Figure S7:** Representative IF images and quantifications (n= 20 for control cells, n=16 for siLMNA cells) of YAP/TAZ subcellular localization in LMNA depleted and control MCF10A cells. Statistical significance was evaluated with one-way ANOVA with Welch correction (p<0.0001). Scale bar 20 μm

**FIGURE S8**

**Figure S8:**  a) Representative 3D reconstruction of MCF10A cells seeded on soft (0,3 kPa) and stiff (40 kPa) static hydrogels. Subnuclear actin fibers and perinuclear microtubules aster sprouting from the microtubule organizing center are highlighted by white arrows in mechano-activated cells. Scale bar 10 μm b) Representative immunofluorescence of microtubule (α-tubulin) and YAP/TAZ in MCF10A seeded on static hydrogels tuned to the indicated stiffnesses. Scale bar 10 μm c) Representative AMOT immunoblot of MCF10A cells seeded on static hydrogels tuned to the indicated stiffnesses. GAPDH serves as loading control. d) Representative immunofluorescence of YAP/TAZ and nuclei in control and AMOT triple KO HEK293 cells seeded in mechano-OFF conditions (dense). Scale bar 20 μm e) Representative AMOT immunoblot of MCF10A cells seeded in mechano-ON conditions and treated with F-actin of microtubules inhibitory drugs (CytochalasinD 1 μM and Nocodazole 5 μM, respectively) for 1hour. GAPDH serves as loading control.
